# A high-quality chromosome-scale assembly of the centipedegrass [*Eremochloa ophiuroides* (Munro) Hack.] genome provides insights into chromosomal structural evolution and prostrate growth habit

**DOI:** 10.1038/s41438-021-00636-6

**Published:** 2021-09-01

**Authors:** Jingjing Wang, Hailing Zi, Rui Wang, Jianxiu Liu, Haoran Wang, Rongrong Chen, Ling Li, Hailin Guo, Jingbo Chen, Jianjian Li, Junqin Zong

**Affiliations:** 1 The National Forestry and Grassland Administration Engineering Research Center for Germplasm Innovation and Utilization of Warm-season Turfgrasses, Institute of Botany, Jiangsu Province and Chinese Academy of Sciences, Nanjing, 210014 China; 2 Novogene Bioinformatics Institute, Beijing, China

**Keywords:** Evolutionary biology, Plant development

## Abstract

Centipedegrass [*Eremochloa ophiuroides* (Munro) Hack.], a member of the Panicoideae subfamily, is one of the most important warm-season turfgrasses originating from China. This grass has an extremely developed prostrate growth habit and has been widely used in transitional and warm climatic regions. To better understand the genetic basis of important biological characteristics, such as prostrate growth and seed yield, in warm-season turfgrasses, we present a high-quality reference genome for centipedegrass and use PacBio, BioNano, and Hi-C technologies to anchor the 867.43 Mb genome assembly into nine pseudochromosomes, with a scaffold N50 of 86.05 Mb and 36,572 annotated genes. Centipedegrass was most closely related to sorghum and diverged from their common ancestor ~16.8 Mya. We detected a novel chromosome reshuffling event in centipedegrass, namely, the nest chromosome fusion event in which fusion of chromosomes 8 and 10 of sorghum into chromosome 3 of centipedegrass likely occurred after the divergence of centipedegrass from sorghum. The typical prostrate growth trait in centipedegrass may be linked to the expansion of candidate *PROSTRATE GROWTH 1* (*PROG1*) genes on chromosome 2. Two orthologous genes of *OsPROG1*, *EoPROG1,* and *EoPROG2*, were confirmed to increase the stem number and decrease the stem angle in *Arabidopsis*. Collectively, our assembled reference genome of centipedegrass offers new knowledge and resources to dissect the genome evolution of Panicoideae and accelerate genome-assisted breeding and improvement of plant architecture in turf plants.

## Introduction

**Fig. 1:**
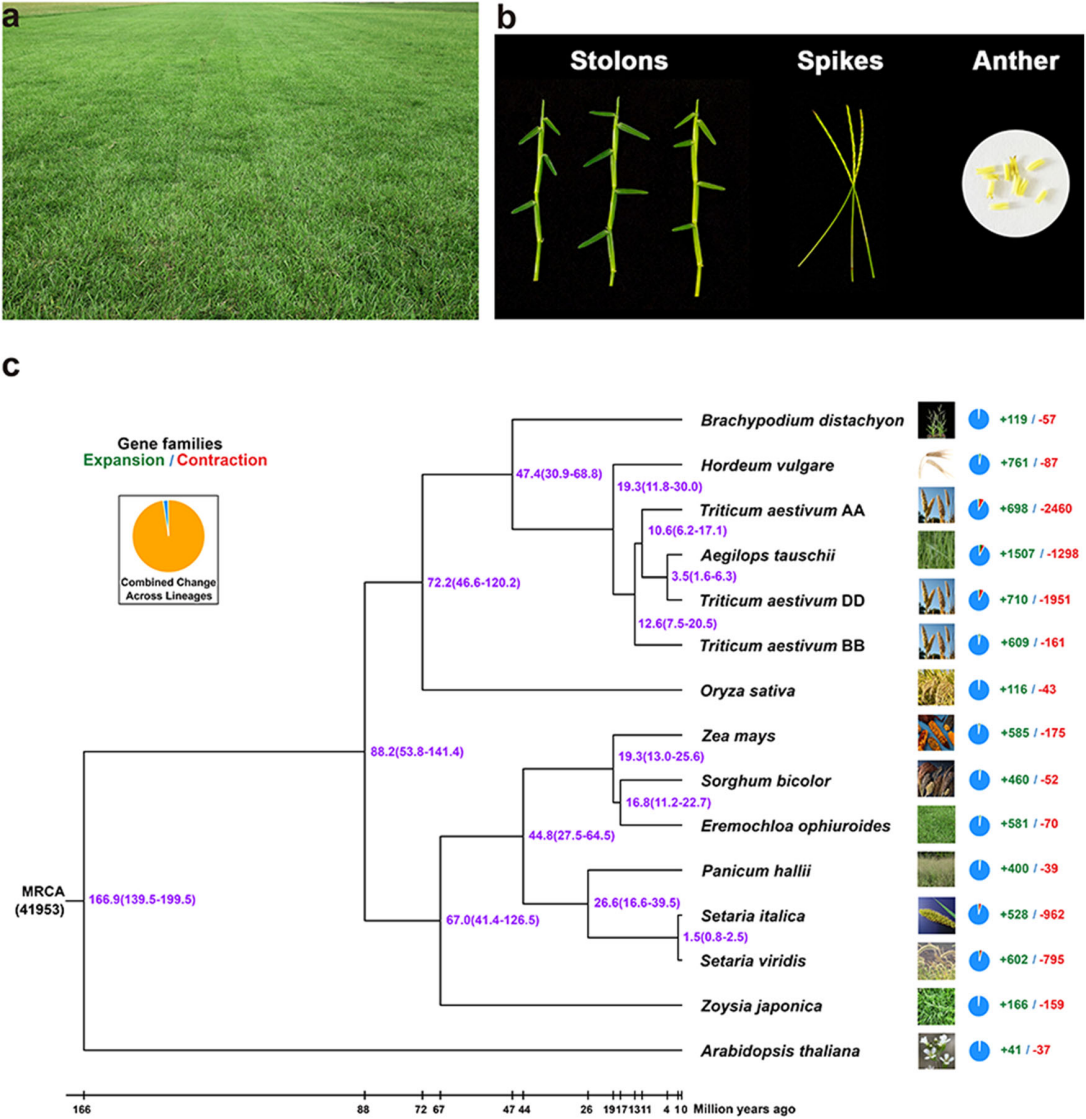
Photographs and comparative genomic analysis of *E. ophiuroides*. **a** Turf performance of *E. ophiuroides*. **b** Stolons, spikes, and anthers of ‘Ganbei’ centipedegrass. **c** Phylogenetic analysis, divergence time, and gene family expansions and contractions among 13 plant species. The phylogenetic tree was constructed on the basis of 1114 single-copy orthologous genes and *A. thaliana* as the outgroup. Divergence times (Mya) are indicated by the purple numbers beside the branch nodes. Gene family expansions and contractions are indicated by green and red numbers, respectively

Centipedegrass [*Eremochloa ophiuroides* (Munro) Hack.] is an indigenous, perennial warm-season (C_4_) grass species in China that is also well adapted for use as turfgrass in transitional and warm climatic regions (Fig. 1a, b). It is now widely used in the southern and eastern USA, Southeast Asia and tropical northern and eastern parts of Australia, and the southern Yangtze River region of China^1,2^. *E. ophiuroides* is well known for good adaptation to infertile and acidic soils and a wide range of climatic conditions^3^. It possesses great potential for use in the turf industry due to its lower management and fertilization requirements compared with other turfgrasses. However, the genomic features that underlie these important biological characteristics are still unclear. Although a low-quality draft genome assembly of *Zoysia* has been released^4^, no high-quality reference genomes representing all warm-season turfgrasses, including important species such as bermudagrass, zoysiagrass, seashore paspalum, and centipedegrass, have been reported to date.

*E. ophiuroides* belongs to the genus *Eremochloa* in the family Poaceae and subfamily Panicoideae^5^. It is a diploid species with a somatic chromosome number of 2*n* = 2*x* = 18^6,7^ and a genome size of ~800 Mb^8^. *E. ophiuroides* is closely related to several main crop species with complex genomes, such as maize (*Zea mays*), sorghum (*Sorghum bicolor*), and foxtail millet (*Setaria italica*). Among these species that also belong to Panicoideae, maize underwent a separate whole-genome duplication (WGD) event, with a chromosome number 2*n* = 2*x* = 20 and a size of ~2.3 Gb^9,10^; the sorghum genome is ~730 Mb in size, with a chromosome number of 2*n* = 2*x* = 20, and includes large transposon structures and substantial genomic rearrangements^11^; and the foxtail millet genome also underwent chromosome reshuffling events, with a genome size of ~423 Mb and chromosome number of 2*n* = 2*x* = 18^12^. Although *E. ophiuroides* presents a comparable genome size to sorghum, its chromosome number is closer to that of foxtail millet. This implies that the *E. ophiuroides* genome most likely formed through a series of genomic events that remain to be determined.

*E. ophiuroides* is the only species in the *Eremochloa* genus that can be used as turfgrass due to its typical prostrate growth habit in the field. During crop domestication, plant architecture was modified in a direction more conducive to increasing crop yield. The transition from prostrate growth to erect growth is one of the most critical events during this process, and genes controlling growth habits play a decisive role in this transition. Previous studies demonstrated that the *PROSTRATE GROWTH 1* (*PROG1*) gene is highly associated with plant architecture changes during domestication in rice and that dysfunction and/or alterations in the expression patterns of *PROG1* have led to erect growth in both Asian and African cultivated rice^13–15^. *PROG1* encodes a C2H2 zinc-finger domain transcription factor located on the short arm of rice chromosome 7 that controls the transition of plant architecture from prostrate growth to erect growth during rice domestication^13,14^. Recently, Wu et al.^16^ found that deletions on rice chromosome 7 eliminating a tandem repeat of zinc-finger genes are closely linked to the role of the *PROG1* gene in plant architecture changes during domestication in both Asian and African rice; they also noted a similar tandem repeat of zinc-finger protein-coding genes and a complex structural variation at the RICE PLANT ARCHITECTURE DOMESTICATION (RPAD) locus in foxtail millet (*S. italica*) and its presumed progenitor, green foxtail (*Setaria viridis*). While erect growth is a more beneficial architecture for most crop plants, prostrate growth is an adaptation to disturbed habitats in wild crop plants and is also the preferred plant architecture for turfgrasses. Considering the extremely developed prostrate growth habit of *E. ophiuroides*, ascertaining the structural characteristics and genome distribution of the *PROG1* gene as well as its expression status in different tissues will contribute to revealing the prostrate growth mechanism of centipedegrass and wild species of crop plants, which will be of great significance for promoting the breeding of turfgrasses and some important crops.

Here, we present a high-quality chromosome-level genome assembly for *E. ophiuroides* obtained via next-generation Illumina, Pacific Biosciences (PacBio), and BioNano sequencing combined with 10× genomic and high-throughput/resolution chromosome conformation capture (Hi-C) technologies. We systematically analyzed the evolutionary position of *E. ophiuroides* in Poaceae, investigated the expansion/contraction of gene families in the *E. ophiuroides* genome assembly through comparative genomic analysis, and proposed a model for the structural evolution of *E. ophiuroides*. The key genes controlling prostrate growth were further screened and analyzed to reveal the typical prostrate growth characteristics of *E. ophiuroides*. Thus, the *E. ophiuroides* reference genome will be a valuable resource for genetic studies and breeding programs in turf plants, both for exploring the genome evolution of Poaceae and the genome-assisted breeding of novel cultivars with desired traits, such as favorable plant architecture traits.

## Materials and methods

### Plant material and genome sequencing and assembly

The sequenced centipedegrass material was a ‘Ganbei’ accession collected from Mount Lushan in Jiangxi Province, China (28°36′N, 116°00′E); this variety has been largely applied in water conservation projects in the lower reaches of the Yangtze River and is preserved in the Grass Research Center of the Institute of Botany, Jiangsu Province and Chinese Academy of Science, China. Leaf tissue of *E. ophiuroides* ‘Ganbei’ was used for Illumina, PacBio, BioNano, and Hi-C library construction. The BioNano library was sequenced on the Saphyr platform. For 10× Genomics sequencing, DNA samples were sequenced on the Illumina HiSeq X Ten platform. The Hi-C library was sequenced on the Illumina NovaSeq PE150 platform. All DNA extraction and sequencing procedures were performed by the Novogene Company (Tianjin, China) (http://www.novogene.com/).

The PacBio reads were used for *de novo* assembly and further polished with Illumina data using Pilon v1.22^17^ to correct indel errors associated with homopolymer repeats in the PacBio data. The sequence consistency and quality of the assembled genome were evaluated using the Burrows-Wheeler Aligner (BWA)^18^ and Benchmarking Universal Single-Copy Orthologs (BUSCO)^19^. To anchor scaffolds onto pseudochromosomes, HiCUP v0.6.1 was used to map and process the reads from the Hi-C library^20^. The genome was divided into bins of equal sizes (500 k), and a contact map plotted with HiCPlotter confirmed the genome structure and quality^21^. The detailed methods are provided in the Supplementary Methods.

### RNA-seq

Root, stem, leaf, node, and spike tissues of the same *E. ophiuroides* ‘Ganbei’ individual were sampled, frozen in liquid nitrogen, and subjected to extraction^22^. Thereafter, cDNA libraries were constructed, and the transcriptomes were sequenced on the Illumina HiSeq 2500 platform by Novogene (Tianjin, China) (http://www.novogene.com/). In addition, these five RNA samples were mixed, and full-length transcriptome sequencing was performed on the PacBio Sequel platform.

### Genome prediction and annotation

Genome prediction and annotation mainly included repeat sequence prediction, gene annotation, and noncoding RNA (ncRNA) prediction. Repeat sequences were predicted through homology searches in the Repbase database (http://www.girinst.org/repbase) by using RepeatMaster and RepeatProteinMask software (http://www.repeatmasker.org/)^23^. The insertion times of the long terminal repeats (LTRs) were estimated using the formula *K*/2*r* (*r* = 1.3 × 10^−8^), considering a higher substitution rate in intergenic regions than in coding regions.

Augustus (http://bioinf.uni-greifswald.de/augustus/), GlimmerHMM (http://ccb.jhu.edu/software/glimmerhmm/) and SNAP (http://homepage.mac.com/iankorf/) were used for de novo gene structure prediction. RNA-seq data from roots, stems, leaves, nodes and spikes were also used to identify gene structures with BLAT (http://genome.ucsc.edu/cgi-bin/hgBlat). In total, 45,472 primitive gene models predicted from the above three prediction methods were corrected with RNA-seq data and integrated into a nonredundant and more complete gene set^24^. Finally, a gene set containing 36,572 gene models was obtained. Furthermore, gene function annotation was performed via BLASTP (*E* value ≤ 1e−5) searches against the NR (https://www.ncbi.nlm.nih.gov/), SwissProt (http://www.UniProt.org/), KEGG (http://www.genome.jp/kegg/) and InterPro (https://www.ebi.ac.uk/interpro/) protein databases.

The noncoding RNA annotations included tRNAs, rRNAs, miRNAs, and snRNAs. tRNAscan-SE (http://lowelab.ucsc.edu/tRNAscan-SE/) was used to search the tRNA sequences in the genome assembly of *E. ophiuroides*. The rRNAs of *E. ophiuroides* were identified by BLAST searches in related species, and the miRNA and snRNA sequences were predicted by applying INFERNAL (http://infernal.janelia.org/) against the Rfam database (v12.0). The detailed methods are provided in the Supplementary Methods.

### Comparative genomic analysis

Genes of thirteen plant species (*E. ophiuroides*, *Aegilops tauschii*, *Brachypodium distachyon*, *Hordeum vulgare*, *Oryza sativa*, *Panicum hallii*, *S. italic*, *S. viridis*, *Z. mays*, *S. bicolor*, *Zoysia japonica*, *Triticum aestivum* and *Arabidopsis thaliana*) were used to analyze gene families. First, the gene sets of each species were filtered by retaining the longest transcript in the coding region and excluding genes encoding proteins with <50 amino acids. Thereafter, OrthoMCL v2.0.9^25^ was used to cluster the filtered gene sets of the thirteen plant species. Through this analysis, we obtained single-copy orthologous groups for phylogenetic analysis and divergence time estimation. A phylogenetic tree was constructed using RAxML with the maximum likelihood-based method and the GTRGAMMA model^26^. Divergence times were predicted using the MCMCtree module of PAML v4.7 (http://abacus.gene.ucl.ac.uk/software/paml.html)^27^ with the following process parameter settings: burn-in = 10,000, sample-number = 100,000 and sample frequency = 2. Furthermore, CAFE (v1.6) (http://sourceforge.net/projects/cafehahnlab/) was used to analyze the expansion and contraction of gene families^28^. The unique, expanded, and contracted gene families of *E. ophiuroides* were annotated via GO and KEGG analyses.

### Resistance (R) gene identification

To identify R genes in the *E. ophiuroides* and *S. bicolor* genomes, HMMER v3.1 with the hidden Markov model (HMM) profile was used to scan for the NB-ARC domain in the Pfam protein family (NB-ARC: PF00931)^29^. The candidate genes containing the nucleotide-binding site (NBS) domain were confirmed in the NCBI Conserved Domain Database (CDD)^30^ and Pfam database^31^, and the genes that did not contain the NBS domain were removed. Each of the candidate genes was checked manually by using available annotations in GenBank to confirm that they encoded the corresponding NBS candidate proteins.

### *EoPROG* gene expression assays and protein structure prediction

Total RNA was extracted from root, stem, leaf, node, and spike tissues using RNA extraction reagent (Vazyme, Nanjing, China). Six *EoPROG* genes, presenting close evolutionary relationships with *OsPROG1* in rice, were subjected to expression assays with quantitative RT-PCR in different tissues. The primers for these genes were designed using Primer 5.0 software, and they are listed in Supplementary Table 1. *EoActin* was used as a housekeeping gene^32^. Furthermore, the protein structures of EoPROG1, EoPROG2, OsPROG1, and Sobic.002G036600.2 were predicted using SWISS-MODEL (https://swissmodel.expasy.org/).

### Ectopic expression of *EoPROG1* and *EoPROG2* in *Arabidopsis*

The coding sequences (CDSs) of *EoPROG1* and *EoPROG2* were first cloned into the pMD19-T vector and then introduced into the pCAMBIA1305.1 vector by LR recombination. The pCAMBIA1305.1 vector contains the hygromycin resistance gene *Hyg*. The pCAMBIA1305.1-*EoPROG* plasmids were introduced into *Agrobacterium tumefaciens* strain *EHA105* and then transformed into the Col-0 ecotype^33^. The transgenic plants were screened by 50 μg mL^−1^ hygromycin and identified using RT-PCR with the primer pair *Hyg*-F/R. The reference sequence was *actin 2* (*AtAct2*, tair: *AT3G18780*). The primers were listed in Supplementary Table 1. The significant difference analysis was performed by SPSS Statistics v.18.0 (Duncan’s test) (SPSS Inc., Chicago, IL, United States).

## Results

### Assembly and annotation

The *E. ophiuroides* genome was assessed by survey analysis, with an estimated genome size, heterozygosity level, and repeat frequency of 856.12 Mb, 0.76% and 59.87%, respectively (Supplementary Table 2, Supplementary Fig. 1a). The assembled genome size was confirmed to be 852.31 Mb, including 640 scaffolds with an N50 of up to 3.67 Mb via next-generation Illumina, Pacific Biosciences (PacBio), and Nanopore sequencing combined with 10× Genomics technologies (Table 1, Supplementary Table 3). In the sequence consistency assessment, the mapping rate of the transcriptome reads in the assembled genome was ~98.87%, and the coverage was ~99.78% (Supplementary Table 4). After quality assessment, the completeness of the gene set in the assembled genome was 95.4% (Supplementary Table 5). These results showed that the assembled *E. ophiuroides* genome presented high sequence consistency and completeness.

**Table 1:** Statistics of assembled *E. ophiuroides*

	Assembly	BioNano assembly	Hi-C assembly
Total length	852.31 Mb	867.40 Mb	867.43 Mb
Contig N50	1.86 Mb	1.86 Mb	1.76 Mb
Scaffold number	640	363	341
Scaffold N50	3.67 Mb	6.10 Mb	86.05 Mb
Scaffold N90	0.91 Mb	1.51 Mb	65.49 Mb
Longest scaffolds	15.67 Mb	18.07 Mb	114.81 Mb

After further improvement of the BioNano assembly, the genome size was confirmed to be 867.40 Mb, including 363 scaffolds, with a scaffold N50 of up to 6.10 Mb (Table 1, Supplementary Table 3). The contigs of the Hi-C library were clustered into 9 pseudochromosomes (Supplementary Table 6, Supplementary Fig. 1b), which was consistent with the results of previous studies. A total of 92.61% of the contigs were assembled into 9 pseudochromosomes, and the scaffold N50 reached 86.05 Mb (Supplementary Table 6, Table 1). The longest and shortest pseudochromosomes were chromosomes 1 and 9, respectively, with sizes of 114.81 and 65.49 Mb (Supplementary Table 6). According to the above analyses, the final assembly of the *E. ophiuroides* genome was 867.43 Mb in size and included 341 scaffolds, with a scaffold N50 of 86.05 Mb (Table 1). The *E. ophiuroides* genome assembly was improved to the chromosome level, with a 92.61% mapping rate on 9 pseudochromosomes.

We identified a total of ~536.07 Mb of repetitive elements, which occupied 61.80% of the genome (Supplementary Table 7). In total, ~528.49 Mb of transposon elements (TEs), which occupied 60.93% of the genome, were annotated (Supplementary Table 8). LTR retrotransposons were the most abundant class of transposons, accounting for 54.26% (~470.69 Mb) of the *E. ophiuroides* genome (Supplementary Table 8). Among these elements, *Ty1/copia* and *Ty3/gypsy* occupied 10.50% and 41.67% of the assembled genome, respectively (Supplementary Table 8). DNA transposons, long interspersed nuclear elements (LINEs) and short interspersed nuclear elements (SINEs) accounted for 4.15, 1.98, and 0.003% of the assembled genome (Supplementary Table 8).

In total, 36,572 genes were identified, with an average length of 3.28 kb and a mean CDS length of 1.14 kb (Supplementary Table 9). The average number of exons per gene and the exon length in the *E. ophiuroides* genome were similar to those in other reported Poaceae plant genomes (Supplementary Table 9). However, the average intron length in the *E. ophiuroides* genome was obviously longer than that in other Poaceae plants (Supplementary Table 9). In addition to protein-coding genes, 2856 miRNA, 616 tRNA, 231 rRNA, and 5174 snRNA genes were identified, accounting for a total of 0.13% of the centipedegrass genome (Supplementary Table 10). The 36,572 identified genes were compared with protein sequences in the NR, SwissProt, KEGG, and InterPro protein databases, and 34,161 (93.4%) genes were annotated in at least one of the protein databases (Supplementary Table 11, Supplementary Fig. 2a). The annotated genes were subjected to BUSCO analysis, and 91.6% completion was achieved.

### Comparative genomic analysis

To reveal the gene family contraction and expansion of *E. ophiuroides*, we compared the gene families of *A. tauschii*, *B. distachyon*, *H. vulgare*, *O. sativa*, *P. hallii*, *S. italica*, *S. viridis*, *Z. mays*, *S. bicolor*, *Z. japonica*, *T. aestivum* (*T. aestivum* AA, *T. aestivum* BB and *T. aestivum* DD), *E. ophiuroides* and *A. thaliana*, which served as an outgroup (Supplementary Table 12). All the genes from these species were clustered into 41,956 gene families, among which 9313 gene families were shared by all Poaceae species. A total of 643 gene families containing 1650 genes appeared to be unique to *E. ophiuroides*. The GO enrichment analysis of unique *E. ophiuroides* genes showed that these genes were associated with 50 GO terms, and the most enriched GO terms were “transferase activity” and “zinc ion binding” (Supplementary Fig. 2b). In addition, KEGG pathway analysis of the unique *E. ophiuroides* genes showed that the most enriched pathways were “base excision repair” and “plant-pathogen interaction” (Supplementary Fig. 2c).

Altogether, 1114 single-copy genes were identified from *E. ophiuroides* and 13 other Poaceae species for phylogenetic analysis (Supplementary Fig. 3a). Our results indicated that *E. ophiuroides* and *S. bicolor* evolved as sister groups that diverged from their common ancestor ~16.8 Mya, and *Z. mays* was identified as sister to the *Eremochloa-Sorghum* lineage (Fig. 1c). This phylogenetic topology was consistent with the traditional classification in which *E. ophiuroides* and *S. bicolor* belong to Andropogoneae and *Z. mays* belong to Maydeae in Panicoideae. To further study the genome evolution of *E. ophiuroides*, we investigated the intracollinearity of *E. ophiuroides* and the collinearity between *E. ophiuroides* and other Poaceae species. By analyzing the paralogous relationships among the nine chromosomes of *E. ophiuroides*, we identified seven major duplications between chromosomes 7 and 5, 3 and 4, 1 and 2, 9 and 2, 3 and 1, 6 and 3, and 8 and 6 and one major duplications within chromosome 1 (Supplementary Fig. 3b).

Furthermore, an analysis of gene family expansion/contraction during the evolution of those 13 plant species was performed based on the phylogenetic relationships. A total of 581 gene families were found to be expanded in centipedegrass, and 70 gene families were contracted (Fig. 1c). The KEGG pathway analysis of the 581 expanded gene families containing 3100 genes showed that these expanded gene families were involved in pathogen resistance-related pathways, among which the most enriched pathway was “plant-pathogen interaction” (Supplementary Fig. 4a). In addition, the GO enrichment analysis showed that these genes were associated with disease resistance, such as “defense response”, “response to stress” and “response to stimulus” (Supplementary Fig. 4b).

After that, we analyzed the shared gene families between *S. bicolor* and *E. ophiuroides* (Fig. 1c). A total of 338 gene families were shared between *E. ophiuroides* and *S. bicolor* only, and 643 gene families appeared to be unique to *E. ophiuroides* (Supplementary Fig. 4c). In addition, the comparison of gene families between *E. ophiuroides* and *S. bicolor* revealed 83 expanded gene families and 56 contracted gene families in *E. ophiuroides*. The above results showed that “plant-pathogen interaction” was the most enriched pathway in unique genes and expanded gene families in *E. ophiuroides*.

Reactive oxygen species (ROS)-related genes, flavonoid-related genes, and plant disease-related R genes play significant roles in detecting pathogen attack and activating the defensive response to pathogens^34–36^. Two upstream regulatory gene families of ROS (https://www.kegg.jp/pathway/map04626) were identified in *E. ophiuroides* and *S. bicolor*, and the gene numbers were similar between these two species (Supplementary Table 13). Moreover, 14 flavonoid-related gene families were identified in *E. ophiuroides* and *S. bicolor*, and the gene numbers of the *cinnamate 4-hydroxylase* (*C4H*), *flavonoid 3’-hydroxylase* (*F3’H*) and *flavonoid 3-O-glucosyltransferase* (*UFGT*) families in *E. ophiuroides* were greater than those in *S. bicolor* (Supplementary Table 13). The best-characterized R genes encode products that contain an NBS domain and a series of leucine-rich repeats (LRRs)^37^. Interestingly, the NBS gene family nearly doubled in *E. ophiuroides* in comparison to *S. bicolor* (597 vs. 337) (Supplementary Table 13, Table 2). The greatest number of NBS genes in *E. ophiuroides* was found on chromosome 4, and their distribution was dispersive (Table 2, Supplementary Fig. 4d). However, the greatest number of NBS genes in *S. bicolor* was found on chromosome 5 (Table 2), and most NBS gene clusters were located at the distal ends of the chromosomes^38^.

**Table 2:** NBS gene numbers in *E. ophiuroides* and *S. bicolor*

*E. ophiuroides*		*S. bicolor*	
Chromosome	NBS gene	Chromosome	NBS gene
Chr1	30	Chr1	18
Chr2	62	Chr2	51
Chr3	71	Chr3	15
Chr4	210	Chr4	8
Chr5	29	Chr5	101
Chr6	14	Chr6	18
Chr7	39	Chr7	31
Chr8	18	Chr8	47
Chr9	60	Chr9	21
–	–	Chr10	26
Other	64	Other	1
Total	597		337

### Differences in the distribution of genes and repeat sequences between *E. ophiuroides* and *S. bicolor*

The *E. ophiuroides* and *S. bicolor* genomes exhibited similar retrotransposon contents (56.24% and 54.52%, respectively)^39^, but the distribution of LTRs on the chromosomes differed between the two species. The LTR density in distal chromosome regions was higher in *E. ophiuroides* than in *S. bicolor*, and the gene density in distal chromosome regions was lower in *E. ophiuroides* (Supplementary Fig. 5). The results indicated that more LTRs were retained in intergenic regions in *E. ophiuroides*, which might be caused by LTR family expansion or a low LTR turnover rate. To reveal the evolution of LTRs in *E. ophiuroides* and *S. bicolor*, we identified full-length LTRs and calculated their insertion times. A total of 21,326 and 19,968 full-length LTRs were identified in *E. ophiuroides* and *S. bicolor*, respectively, and LTRs underwent expansion in *E. ophiuroides* ~2 million years ago, while most LTR insertions in *S. bicolor* occurred <1 million years ago (Supplementary Fig. 6a).

The *athila*, *del* and *tat* subfamilies of the *Ty3/gypsy* elements and the *sire* subfamily of the *Ty1/copia* elements were the most enriched retrotransposon classes in *E. ophiuroides* and *S. bicolor* (Supplementary Table 14). The insertion times of the *del* elements were similar between *E. ophiuroides* and *S. bicolor*, while the other three subfamilies all showed earlier insertions in *E. ophiuroides* (Supplementary Fig. 6b). We also illustrated the distribution of *athila* and *del* elements on the chromosomes. *del* elements distributed in distal and proximal chromosome regions in *E. ophiuroides* and *S. bicolor*. *athila* elements were rarely found in gene-rich regions in *S. bicolor* but were distributed in distal regions of the *E. ophiuroides* chromosomes (Supplementary Fig. 5). These results implied that *athila* elements have been inserted and maintained in the *E. ophiuroides* genome for ~2 million years. The formation of solo-LTRs through unequal homologous recombination counterbalances the amplification of LTR retrotransposons^40^. The retention of *athila* elements in *E. ophiuroides* is likely due to their perennation and vegetative propagation characteristics, which reduce the recombination rate.

### Reshuffling and structural evolution of *E. ophiuroides* chromosomes

**Fig. 2:**
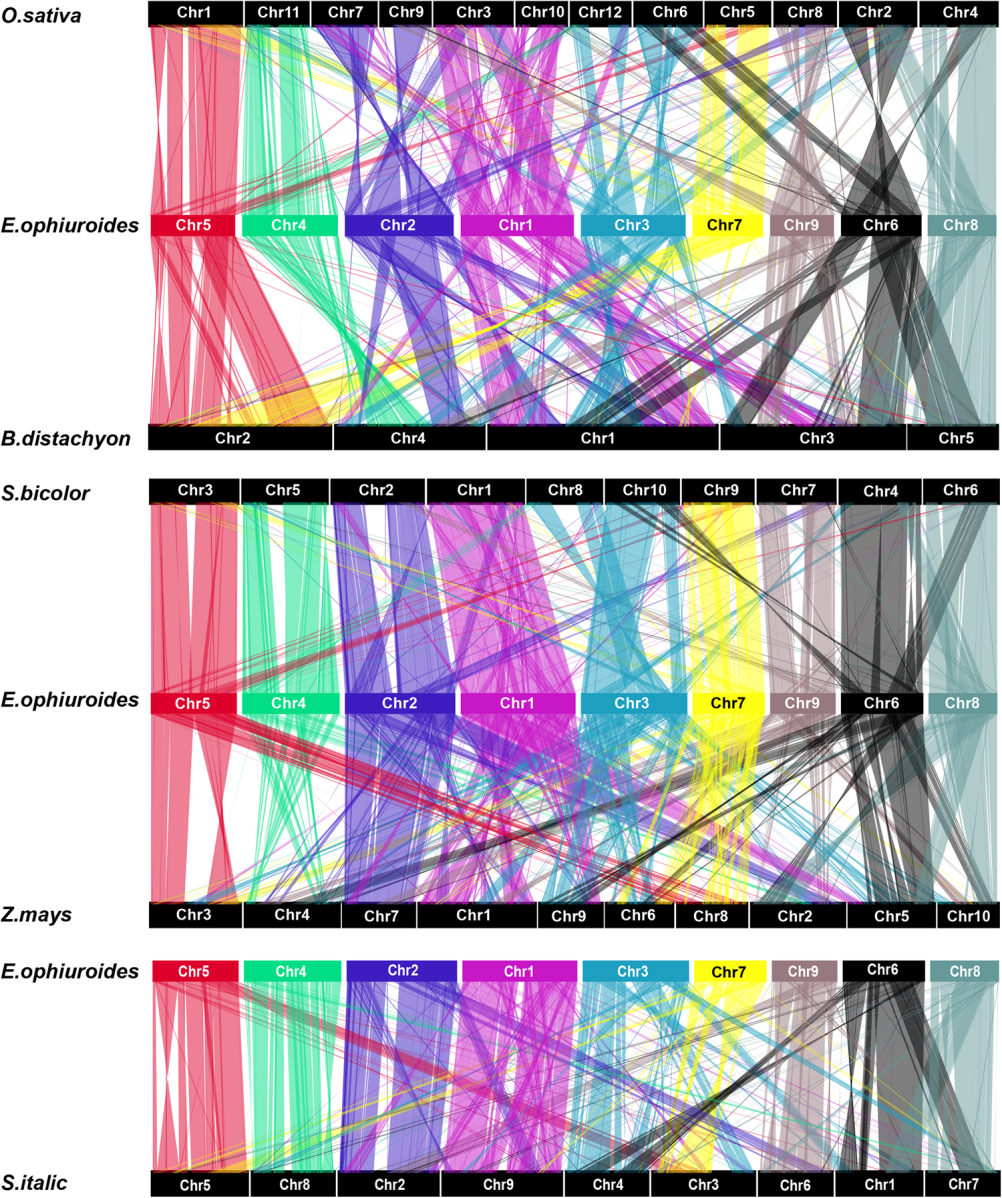
Chromosome analyses of *E. ophiuroides*. Syntenic blocks between *E. ophiuroides* and other sequenced grass genomes, including *O. sativa*, *B. distachyon, S. bicolor, Z. mays,* and *S. italica*

It has been reported that the chromosomes of grasses undergo extensive rearrangements despite the close evolutionary relationships among grasses^12,41^. Previous studies have revealed that the ancestor of grasses experienced one WGD and two nest chromosome fusion events, producing 12 common chromosomes^12,41,42^. To investigate the evolution of the *E. ophiuroides* chromosomes, the genomes of *E. ophiuroides*, *B. distachyon*, *O. sativa*, *S. bicolor*, *Z. mays*, and *S. italica*, belonging to the three subfamilies of grasses (Pooideae, Ehrhartoideae, and Panicoideae), were chosen for comparative analyses. Massive collinear blocks were identified among these grasses, which indicated well-preserved genome structures in this family. A total of 45.10% (15,620 gene pairs), 46.39% (16,066 gene pairs), 58.36% (20,212 gene pairs), 51.72% (17,911), and 50.33% (17.430) of the *E. ophiuroides* genome was syntenic with *B. distachyon*, *O. sativa*, *S. bicolor*, *Z. mays*, and *S. italica*, respectively (Fig. 2, Supplementary Table 15).

Using the rice genome as the reference comparison strain genome because of its 12 retained chromosomes, we found that *E. ophiuroides* chromosome 1 is largely collinear with rice chromosomes 3 and 10, *E. ophiuroides* chromosome 2 is collinear with rice chromosomes 7 and 9, and *E. ophiuroides* chromosome 3 is collinear with rice chromosomes 3 and 10 (Fig. 2). In addition, large regions of collinearity were identified between *E. ophiuroides* chromosome 6 and rice chromosome 2 and between *E. ophiuroides* chromosome 9 and rice chromosome 8, while small regions of collinearity were detected between *E. ophiuroides* chromosome 6 and rice chromosomes 6 and 4 and between *E. ophiuroides* chromosome 9 and rice chromosomes 9 and 4 (Fig. 2). This result demonstrated that complex chromosome rearrangements as well as chromosome fusions have occurred among the *E. ophiuroides* chromosomes. In addition to the three nest chromosome fusions that occurred in *E. ophiuroides* (chromosomes 1, 2, and 3), two and three nest chromosome fusions were identified in the *S. bicolor* and *S. italica* genomes, respectively (Fig. 2). Considering the close relationship between *S. bicolor* and *E. ophiuroides*, a further comparative analysis of collinearity was performed between them. We noted that *E. ophiuroides* chromosome 3 was highly collinear with *S. bicolor* chromosomes 8 and 10 (Fig. 2), indicating that *S. bicolor* chromosomes 8 and 10 might have fused during their evolution, leading to the difference in chromosome number between *S. bicolor* and *E. ophiuroides*. A similar chromosome fusion event associated with high collinearity between chromosome 3 of *S. italica* and chromosomes 8 and 9 of *S. bicolor* was also detected^12^.

**Fig. 3:**
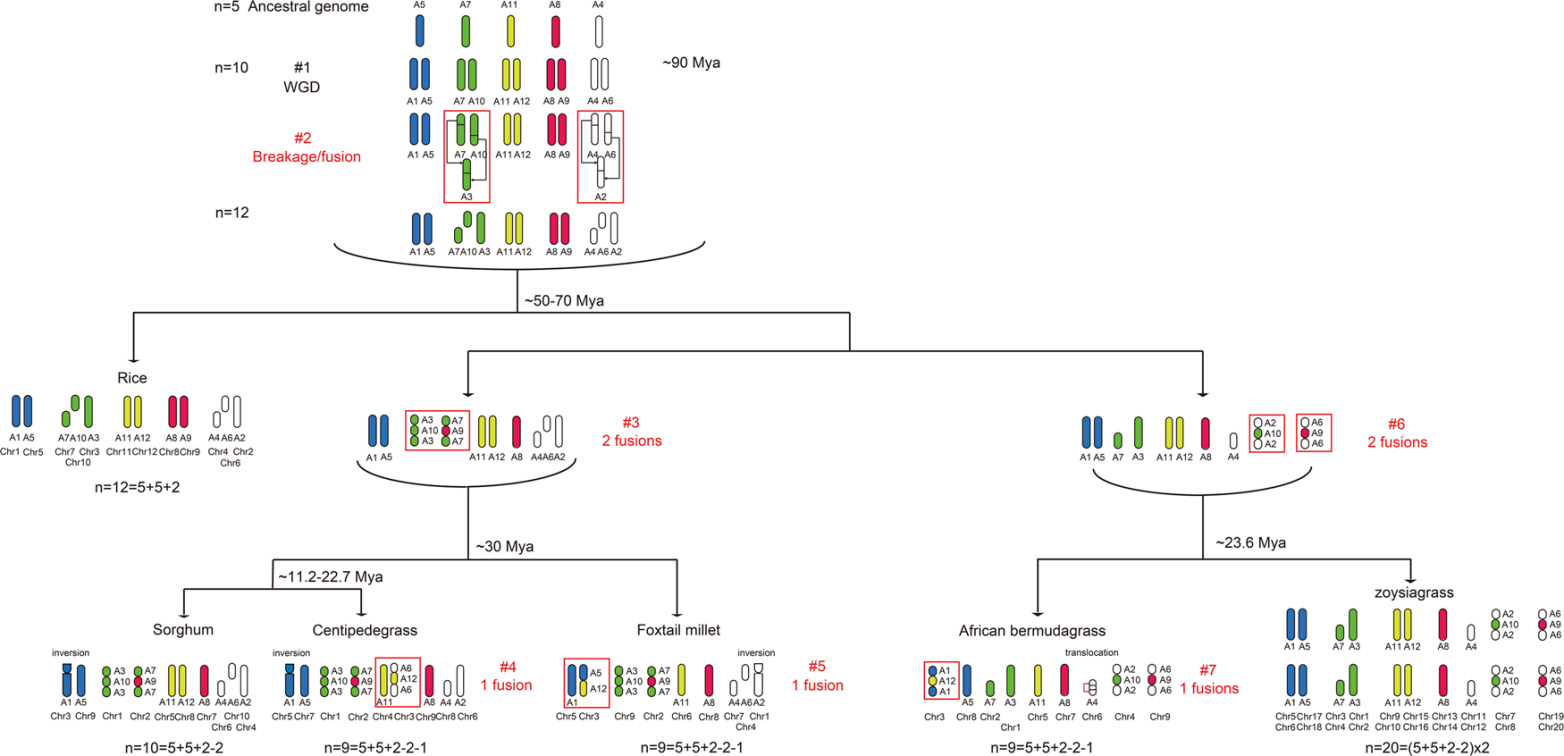
A model of chromosome structural evolution of grasses. A model of chromosome structural evolution in the centipedegrass, rice, sorghum, foxtail millet, African bermudagrass, and zoysiagrass genomes starting from a common ancestor with *n* = 5 chromosomes

The phylogenetic relationships among the grasses were refined and showed that *E. ophiuroides* split from *S. bicolor*, which occurred after the divergence of *S. italica* (Fig. 3). By combining the data obtained in the current study with previously reported genome data from rice^41^, sorghum^43^, and foxtail millet^12^, we performed a detailed analysis of the common and lineage-specific patterns of conservation between those different genomes. Accordingly, we proposed a model (Fig. 3) of the structural evolution of *E. ophiuroides* and three other grass species from a common ancestor with a basic number of five chromosomes (A5, A7, A11, A8, and A4), including orthologous chromosomes that exhibit shared ancestral duplications. In rice, sorghum, and foxtail millet, the shared duplications corresponding to ancestral chromosomes A5, A7, A11, A8, and A4 were congruent with those reported previously by Salse et al.^41^ and Zhang et al.^12^. In *E. ophiuroides*, a duplication corresponding to ancestral chromosome A5 was found between chromosomes 5 and 7; a duplication corresponding to A7 was found on chromosomes 1 and 2; ancestral chromosome A11 corresponded to conserved chromosomes 4 and 3; a duplication corresponding to A8 was detected between chromosomes 9 and 2; and a duplication corresponding to A4 was observed on orthologous chromosomes 3, 8, and 6 (Fig. 3).

As described in the previous studies^12,41,42,44^, an intermediate ancestor with *n* = 12 (5 + 5 + 2) chromosomes evolved from a common ancestor with a genome including five chromosomes (A5, A7, A11, A8, and A4) after a single WGD (event #1) and two nest chromosome fusion events (event #2) observed in grass species (Fig. 3). Subsequently, rice retained the basic structure of 12 chromosomes, although additional segmental duplications occurred in its genome. In the ancestral genome with 12 chromosomes, the occurrence of two chromosomal fusions (between A3 and A10 and between A7 and A9) resulted in another intermediate ancestor of sorghum, foxtail millet, and centipedegrass with n=10 chromosomes (5 + 5 + 2 − 2) (event #3) (Fig. 3). Thereafter, the sorghum genome structure remained similar to that of the ancestral genome with ten chromosomes (5 + 5 + 2 − 2), while the centipedegrass genome underwent an additional chromosome fusion (between A6 and A12) (event #4), as did foxtail millet (between A5 and A12) (event #5) (Fig. 3). Thus, similar to foxtail millet, centipedegrass evolved independently from the ancestor with *n* = 10 chromosomes and finally developed a genome structure with 9 chromosomes (*n* = 9 = 5 + 5 + 2 − 2 − 1) (Fig. 3). Compared with sorghum, foxtail millet, and centipedegrass, two chromosomal fusions occurred in the intermediate ancestor of African bermudagrass and zoysiagrass (between A2 and A10 and between A6 and A9) (event #6) (Fig. 3). After that, the African bermudagrass genome underwent additional chromosome fusion (between A1 and A12) (event #7) and chromosome translocation in A4 (Fig. 3).

### The prostrate growth habit of *E. ophiuroides*

**Fig. 4:**
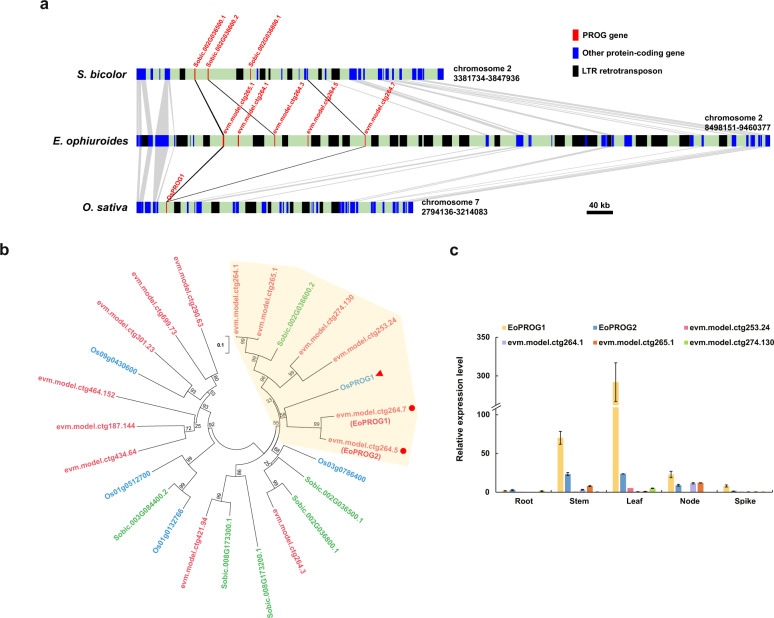
Screening and analysis of candidate *PROG* genes for prostrate growth. **a** Collinearity analysis of chromosome segments containing *PROG* homologs in *O. sativa*, *S. bicolor,* and *E. ophiuroides*. The red, blue, and black boxes indicate *PROG* genes, other protein-coding genes, and LTR retrotransposons, respectively. The gray region represents the shared collinear sequence. Black lines indicate the orthologous relationships of *PROG* genes. **b** Phylogenetic tree of PROG proteins. The gene IDs in blue represent the *OsPROG1* gene (indicated by the red triangle) and its homologs in *O. sativa*. The gene IDs in green represent the homologs of the *OsPROG1* gene in *S. bicolor*. The gene IDs in red represent the homologs of the *OsPROG1* gene in *E. ophiuroides*. The red dots indicate the closest genetic relationships between candidate *PROG* genes and *OsPROG1* in rice. The yellow area shows that one homologous gene in *S. bicolor* and six homologous genes in *E. ophiuroides* exhibited close genetic relationships with *OsPROG1* in rice. **c** The expression patterns of six candidate *PROG* genes of *E. ophiuroides* in roots, stems, leaves, nodes, and spikes

Compared with domesticated grain crops, *E. ophiuroides* retains its prostrate growth habit. It has been reported that the prostrate growth of wild rice from Yuanjiang County in China is controlled by a semidominant gene, *OsPROG1* (*Os07g0153600*), on chromosome 7, which encodes a single Cys(2)-His(2) zinc-finger protein^13^. Inactivating *OsPROG1* function can lead to erect growth and an increased grain number^13^. The NCBI database shows that the preferred name of the *OsPROG1* gene of rice is *zinc finger protein GIS3* (*GLABROUS INFLORESCENCE STEMS 3*), and there are 5 *GIS3* genes in *O. sativa*. According to these sequences, we identified six and 14 *GIS3* homologs in *S. bicolor* and *E. ophiuroides*, respectively (Supplementary Table 16). Furthermore, three *GIS3* homologs in *S. bicolor* were located on chromosome 2, and eight *GIS3* homologs in *E. ophiuroides* were also located on chromosome 2 (Supplementary Table 16). Chromosome 2 of *S. bicolor* was highly collinear with chromosome 2 of *E. ophiuroides* (Fig. 2). Previous studies have shown that chromosome 2 of *S. bicolor* is collinear with chromosomes 7 and 9 of *O. sativa* and that the terminal sequences of chromosome 2 of *S. bicolor* aligned with those of chromosome 7 of *O. sativa*^39^. The collinearity analysis of chromosome segments containing *OsPROG1* genes in *O. sativa* and *PROG* homologs in *S. bicolor* and *E. ophiuroides* showed an obvious series of *PROG* homologs in *E. ophiuroides*, resulting in the formation of gene clusters (Fig. 4a). These results showed obvious expansion of *PROG* homologs on chromosome 2 in the *E. ophiuroides* genome.

The phylogenetic tree showed that 1 homolog (*Sobic.002G036600.2*) in *S. bicolor* and 6 homologs (*evm.model.ctg264.7*, *evm.model.ctg264.5*, *evm.model.ctg264.1*, *evm.model.ctg265.1*, *evm.model.ctg274.130* and *evm.model.ctg253.24*) in *E. ophiuroides* showed close evolutionary relationships with *OsPROG1* in rice (Fig. 4b). Among these genes, *evm.model.ctg264.7* (*EoPROG1*) and *evm.model.ctg264.5* (*EoPROG2*) exhibited the closest genetic relationship with *OsPROG1* in rice (Fig. 4b). Amino acid sequence alignment showed that these genes shared a conserved C2H2-type zinc-finger domain (Supplementary Fig. 7a). According to quantitative RT-PCR data from various *E. ophiuroides* tissues (root, stem, leaf, node, and spike), *EoPROG1* and *EoPROG2* both showed high expression levels in stems and leaves (Fig. 4c). Protein structure prediction showed that these four proteins exhibited similar structures (Supplementary Fig. 7b).

**Fig. 5:**
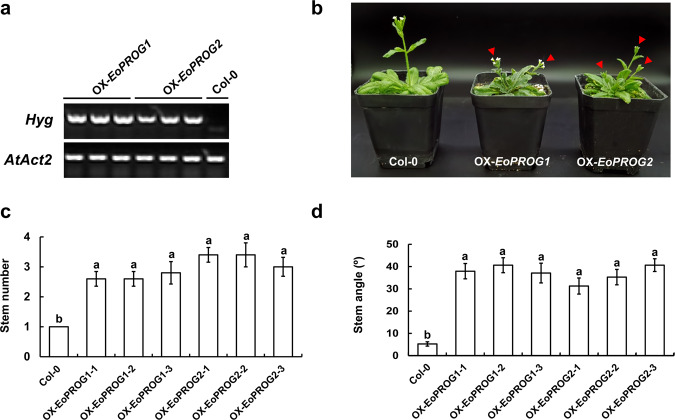
Ectopic expression of *EoPROG1* and *EoPROG2* in Col background *Arabidopsis* (Col-0). **a** RT-PCR analyses of T3-overexpressing *EoPROG1* and *EoPROG2* transgenic lines. The reference gene is *AtAct2*. The resistance marker gene is *Hyg*. **b** Phenotypes of 48-day-old wild-type (Col-0) and T_3_ transgenic plants. **c** The stem number of wild-type (Col-0) and T_3_ transgenic plants. **d** The stem angle of wild-type (Col-0) and T_3_ transgenic plants. Data are the means (*n* = 10), with error bars showing the standard deviation. Letters above bars indicate significant differences between the respective values (*p* < 0.05)

To investigate the function of *EoPROGs*, the CDSs of *EoPROG1* and *EoPROG2* were driven by the lac promoter and introduced into *Arabidopsis*. In total, eight hygromycin (Hyg)-resistant T_1_ transgenic lines of *EoPROG1* and four T_1_ transgenic lines of *EoPROG2* were obtained. Three T_3_ transgenic lines of *EoPROG1* and *EoPROG2* were identified with RT-PCR, and the *EoPROG1* and *EoPROG2* transgenic lines showed similar phenotypes (Fig. 5a, b). Compared with Col-0 plants, overexpressing *EoPROG1* and *EoPROG2* influenced the erect growth of *Arabidopsis* (Fig. 5b). The stem number of the *OX-EoPROG* transgenic lines was obviously greater than that of the Col-0 plants (Fig. 5c). Moreover, the stem angle of the *OX-EoPROG* transgenic lines was significantly higher than that of Col-0 plants (Fig. 5d). These results indicated that *EoPROG1* and *EoPROG2* might have significant functions in regulating the prostrate growth of *E. ophiuroides*.

## Discussion

### Chromosome structural evolution in *E. ophiuroides*

In our study, massive collinear blocks were detected by intergenic analyses between centipedegrass, brachypodium, rice, sorghum, maize, and foxtail millet, which revealed a close evolutionary relationship among these grass species. However, the apparent difference in genome size and chromosome numbers between centipedegrass and other grass species implied that centipedegrass chromosomes may have undergone different rearrangements from other species during its evolution. It is known that extensive chromosome rearrangements occurred in grass species despite their close evolutionary relationships^12,41,45^, and different grass species evolved from the first intermediate ancestor with 12 common chromosomes, which were derived from one WGD and two nest chromosome fusion events in the ancestor of the grasses^12,41,42^. Through a detailed comparative genomic analysis, we inferred that centipedegrass diverged from the second intermediate ancestor with *n* = 10 chromosomes, which was formed by two chromosomal fusions (between A3 and A10 A7 and A9) of the first intermediate ancestor. Interestingly, since Zhang et al.^12^ reported that foxtail millet chromosome 3 was collinear with ancestral chromosomes A5 and A12, we also found that centipedegrass chromosome 3 was homologous to ancestor chromosomes A6 and A12 in this study (Fig. 3). This finding indicated that two chromosomes (A6 and A12 or A5 and A12) of the ancestor might have fused into one chromosome corresponding to chromosome 3 in centipedegrass or foxtail millet. Since sorghum retains a similar chromosome constitution as the intermediate ancestor possessing *n* = 10 chromosomes, the occurrence of this extra chromosome fusion might be the major driving force for the formation of different genome structures in centipedegrass and foxtail millet. Furthermore, high collinearity between centipedegrass chromosome 3 and sorghum chromosomes 8 and 10 indicated that centipedegrass chromosome 3 resulted from the fusion of sorghum chromosomes 8 and 10, which most likely occurred after the divergence of centipedegrass from sorghum. A similar chromosome rearrangement was also discovered in foxtail millet^12^.

African bermudagrass and zoysiagrass are both warm-season turfgrasses classified in the Chloridoideae subfamily^4,46^; they differ from centipedegrass belonging to the Panicoideae subfamily. The chromosome rearrangement analysis showed that African bermudagrass and zoysiagrass had different intermediate ancestors with two chromosomal fusions (between A2 and A10 and between A6 and A9), while sorghum, centipedegrass and foxtail millet had the same intermediate ancestor as the other two chromosomal fusions (between A3 and A10 and between A7 and A9) (Fig. 3). These results showed that the different chromosomal fusion methods were related to the differentiation of different subfamilies. On that basis, the African bermudagrass genome underwent additional chromosome fusion (between A1 and A12) and chromosome translocation in A4 (Fig. 3), indicating that chromosome fusion events may have caused genomic variation and contributed to the speciation of grass plants.

### Prostrate growth habit of *E. ophiuroides*

Among the three nest chromosome fusion events identified between *E. ophiuroides* and *O. sativa*, the fusion of chromosomes 7 and 9 of *O. sativa* to produce chromosome 2 of *E. ophiuroides* might have an important impact on the centipedegrass prostrate growth habit. In wild rice, the semidominant gene *PROG1* on chromosome 7 has been reported to affect prostrate growth, and the inactivation of *PROG1* function can lead to erect growth and more grains^13^. The *PROG1* gene in rice encodes a single C2H2 zinc finger protein^13^ with the preferred name *zinc finger protein GIS3* according to the NCBI database. In Asian cultivated rice *O. sativa*, a 110 kb deletion on the short arm of chromosome 7, which contains a tandem repeat of seven zinc finger genes, is closely linked to the *OsPROG1* gene^16^. Deletions of the three zinc finger genes mentioned above have been verified to regulate the prostrate growth of wild rice^16^. In addition, a similar tandem repeat of a zinc finger protein-coding gene deletion is observed in African cultivated rice, indicating that the zinc finger gene cluster may exhibit the conserved function of regulating prostrate plant growth in the Poaceae family^16^. In African cultivated rice (*Oryza glaberrima*), the *PROG7* gene, which is identical to *OsPROG1*, was verified to be necessary for the prostrate growth of African wild rice (*Oryza barthii*)^47^. The expression pattern showed that *ObPROG7* had high expression levels in young leaves, leaf sheaths, and shoot apical meristems^47^. *PROG* homologs exhibited obvious expansion on chromosome 2 of the *E. ophiuroides* genome, and their locations were focused at the chromosome 2 termini (Fig. 4a). In addition, a large number of LTR retrotransposons appeared near *PROG* homologs, indicating that *PROG* gene expansion might be closely associated with LTR retrotransposon insertion (Fig. 4a). Therefore, the *PROG* homologs on chromosome 2 of the *E. ophiuroides* genome may serve significant functions in the prostrate growth of centipedegrass.

The phylogenetic tree showed that 1 *PROG* homolog (*Sobic.002G036600.2*) in sorghum and 6 *PROG* homologs (*evm.model.ctg264.7*, *evm.model.ctg264.5*, *evm.model.ctg264.1*, *evm.model.ctg265.1*, *evm.model.ctg274.130* and *evm.model.ctg253.24*) in centipedegrass exhibited close genetic relationships with *OsPROG1* in rice (Fig. 4b). Among these genes, *EoPROG1* and *EoPROG2* showed the closest genetic relationship with *OsPROG1* (Fig. 4b). Both *EoPROG1* and *EoPROG2* exhibited high expression levels in the stems and leaves of centipedegrass, which is consistent with the expression pattern of *ObPROG7* in African wild rice (Fig. 4c). Furthermore, *EoPROG1* and *EoPROG2* presented similar protein structures to OsPROG1 in rice (Supplementary Fig. 7b). The transgenic results showed that overexpressing *EoPROG1* and *EoPROG2* in *Arabidopsis* increased the number and angle of stems (Fig. 5c, d), which was consistent with previous research results in rice^16^. These results showed that *EoPROG1* and *EoPROG2* might be the most important genes in centipedegrass prostrate growth regulation.

The generation of a reference genome sequence of *E. ophiuroides* fills the sequencing gap in *Eremochloa* genus plants, enriches the available turfgrass genome sequences, and provides resources for centipedegrass molecular breeding. The comparative analysis of *E. ophiuroides* genomes allows us to better understand chromosome evolution in Poaceae and has great significance for our understanding of turfgrass prostrate growth.

## Supplementary information

Supplementary materials

Supplementary Table 15

Supplementary method

## Data Availability

The genomic raw data in this study can be found in the NCBI repository http://www.ncbi.nlm.nih.gov/bioproject/PRJNA682293. The assembly and annotation data in this study can be found at https://figshare.com/s/8256acffdb73bb050045.
