# Supplementary material for: A high-quality chromosome-scale assembly of the centipedegrass [Eremochloa ophiuroides (Munro) Hack.] genome provides insights into chromosomal structural evolution and prostrate growth habit
Source: Hortic Res. 2021 Sep 1;8:201. doi: 10.1038/s41438-021-00636-6 (PMC8408263; doi:10.1038/s41438-021-00636-6)
Supplement: Supplementary file 1 — Supplementary materials [file 41438_2021_636_MOESM1_ESM.docx]

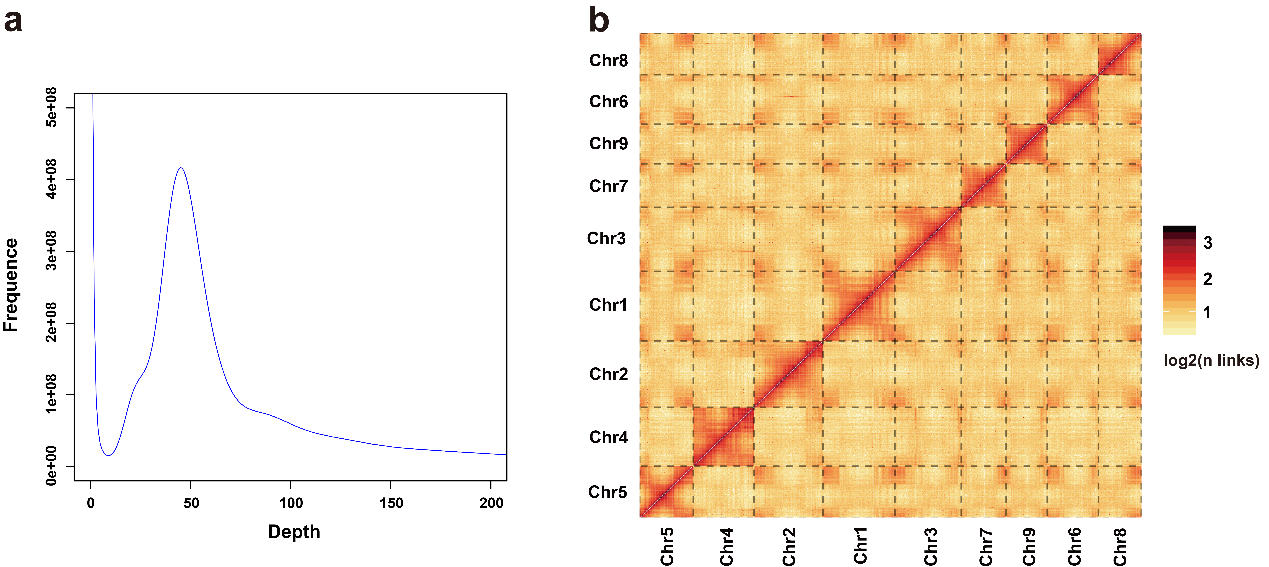


**Supplementary Fig. 1. Survey analysis and heat map of the Hi-C chromosome data of *E. ophiuroides*.** **(a)** The distribution of the *E. ophiuroides* 17-mer frequency, with the main peak at 44. **(b)** Heat map of chromatin contact matrices generated by aligning a Hi-C dataset to the *E. ophiuroides* genome.


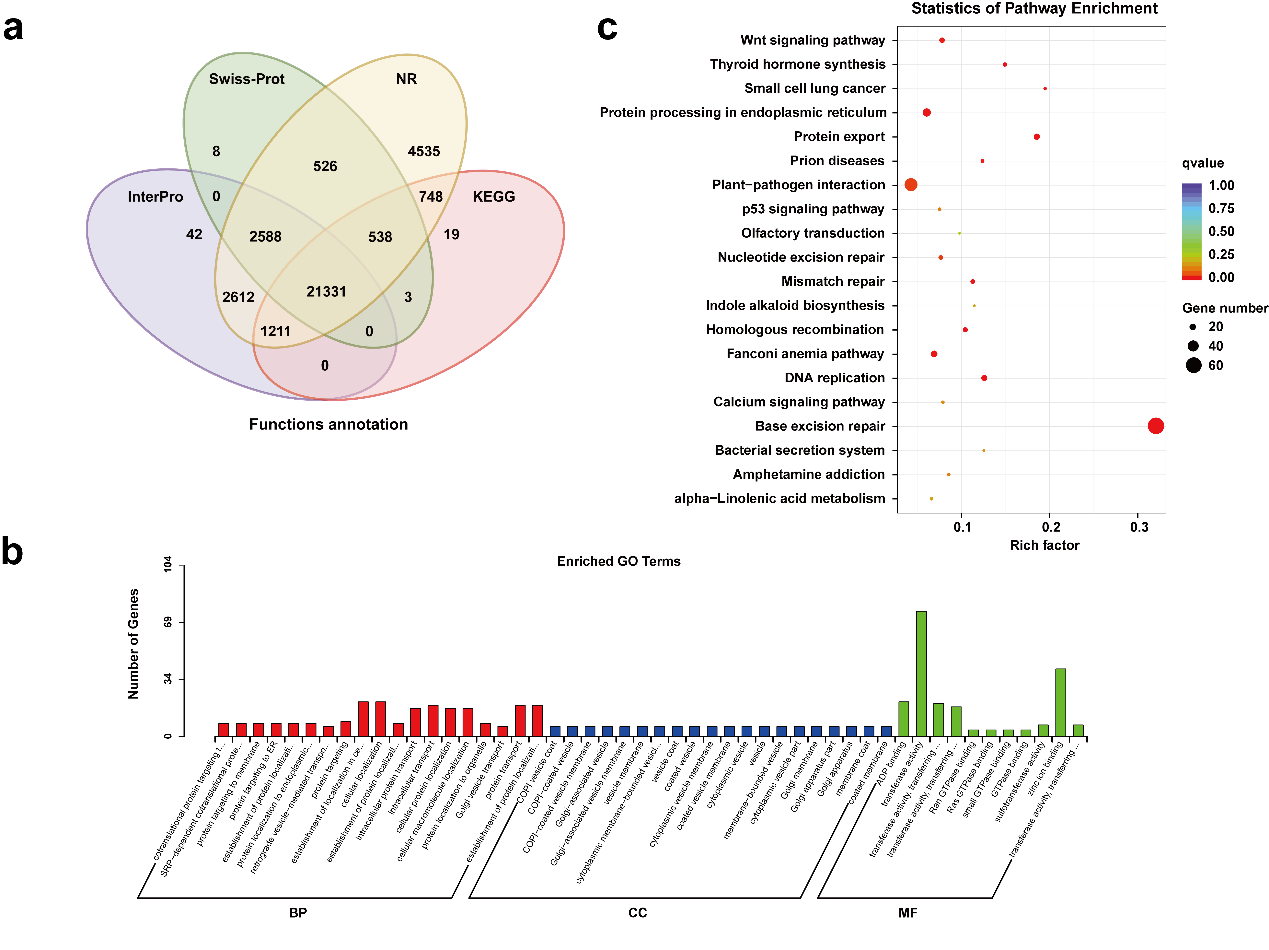


**Supplementary Fig. 2. *E. ophiuroides* gene annotation and unique gene analyses. (a)** Venn diagram analysis of the number of *E. ophiuroides* genes annotated in the NR, SwissProt, KEGG and InterPro protein databases. **(b)** GO enrichment analysis of unique *E. ophiuroides* genes. **(c)** KEGG pathway analysis of unique *E. ophiuroides* genes.


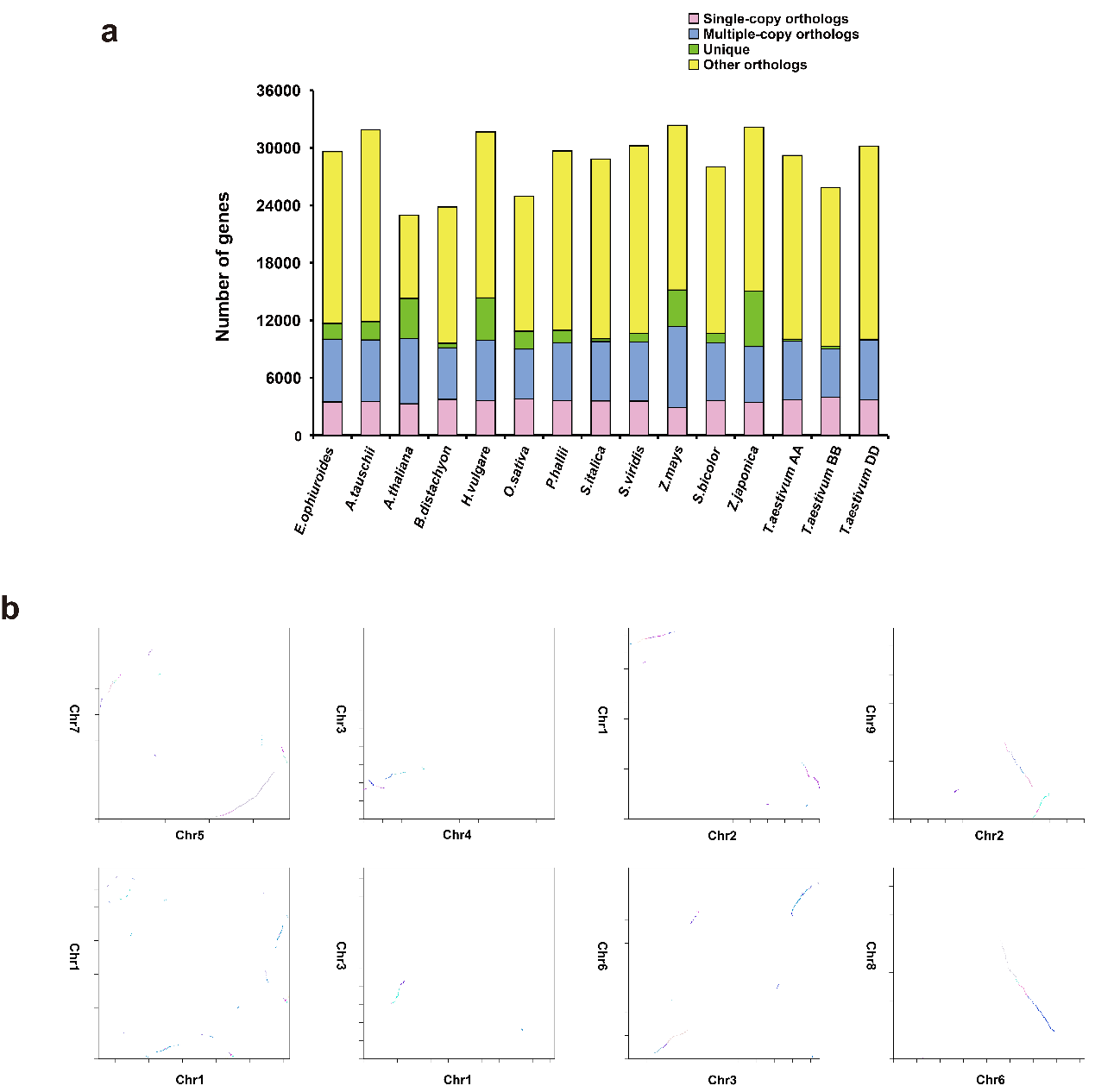


**Supplementary Fig. 3. Comparative genomic analyses and synteny blocks within *E. ophiuroides*. (a)** Clusters of orthologous and paralogous genes in *E. ophiuroides* and other sequenced Poaceae plants. **(b)** The gene set of *E. ophiuroides* was compared to itself, and paralogous genes in the *E. ophiuroides* genome were thus identified. Synteny blocks were found between chromosomes 7 and 5, 3 and 4, 1 and 2, 9 and 2, 3 and 1, 6 and 3, and 8 and 6 and even within chromosome 1, which resulted from the ancient whole-genome duplication.


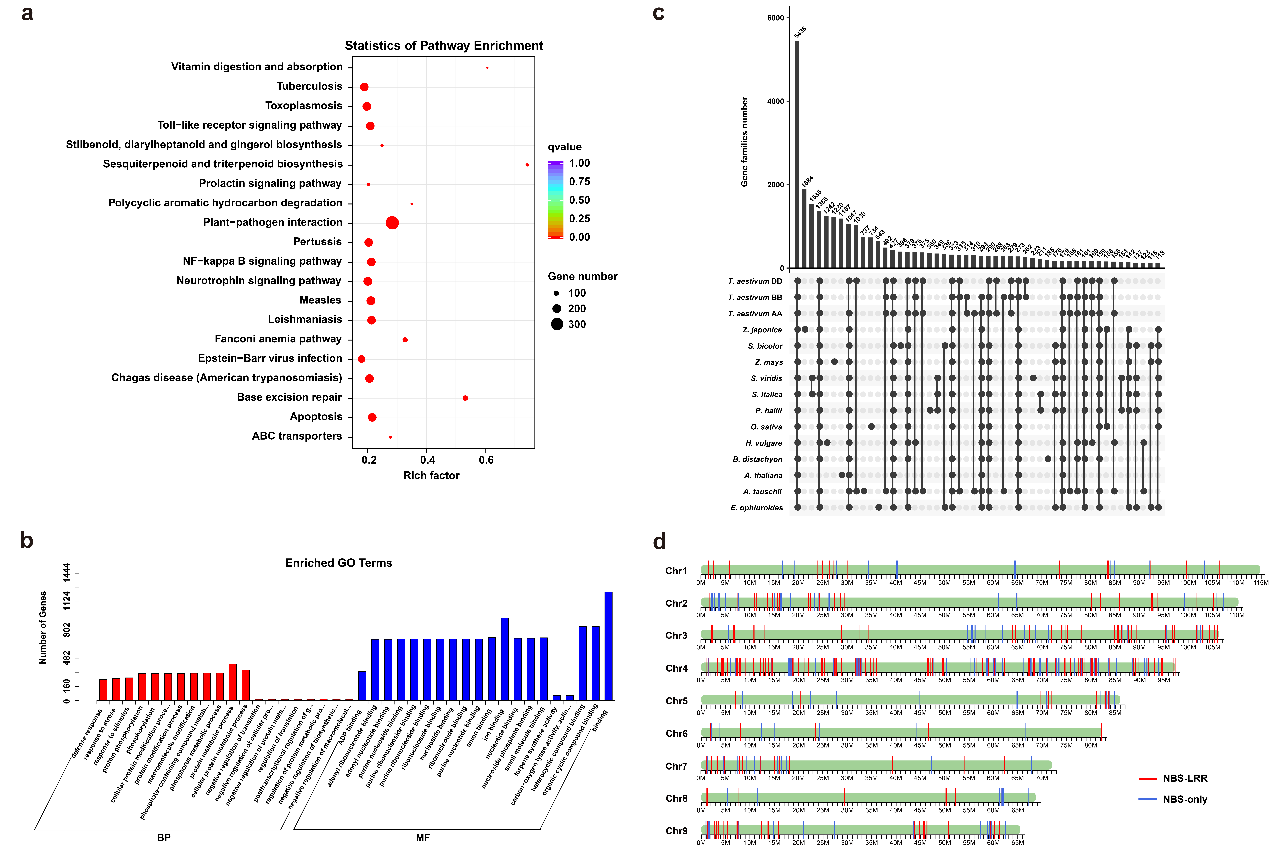


**Supplementary Fig. 4. Comparative genomic analyses. (a)** KEGG analysis of expanded gene families in the *E. ophiuroides* genome. **(b)** GO enrichment analysis of expanded gene families in the *E. ophiuroides* genome. **(c)** Analysis of the number of common and unique gene families. **(d)** The distribution of NBS genes on each chromosome of *E. ophiuroides*. Red: NBS gene with an LRR domain; Blue: NBS gene without an LRR domain.


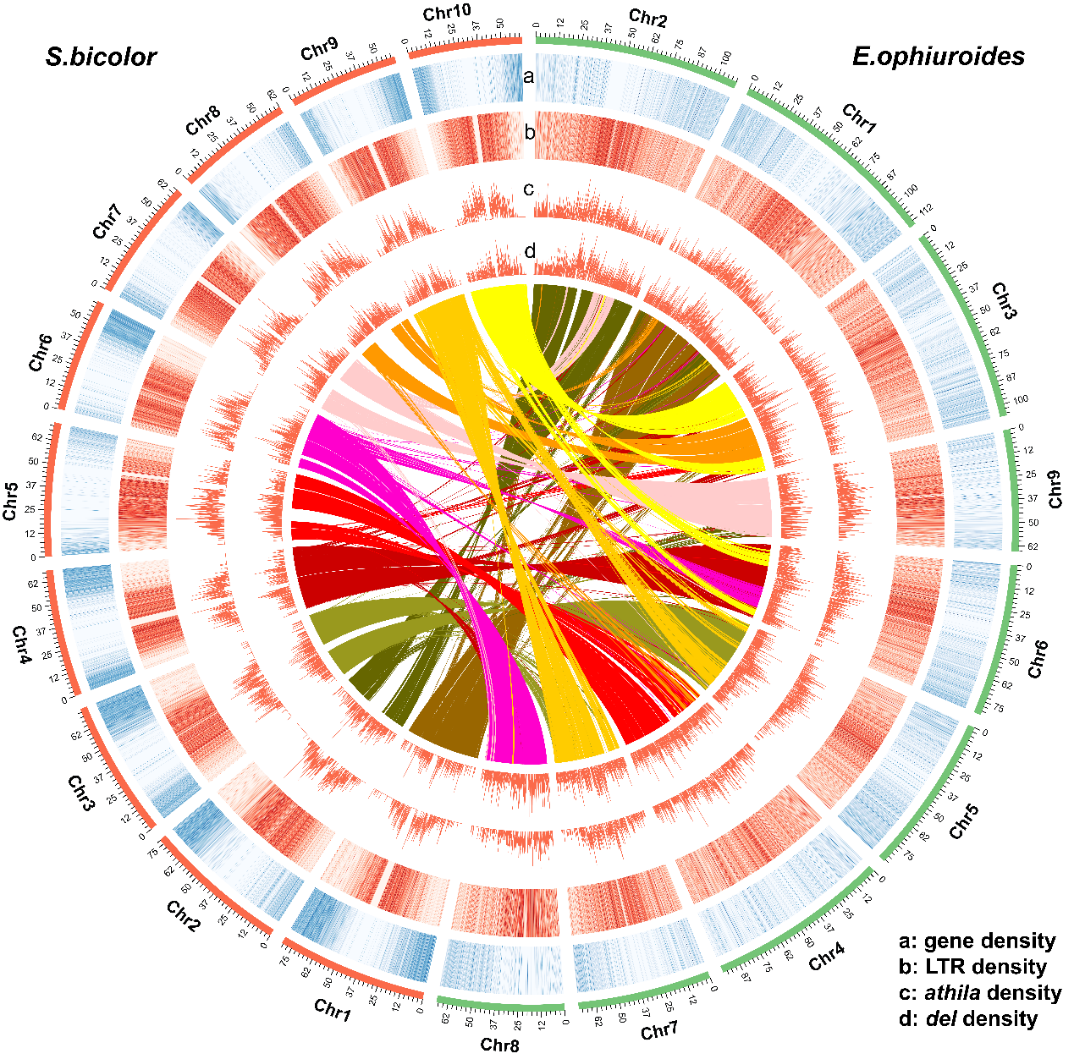


**Supplementary Fig. 5. Chromosomal feature analyses between the *E. ophiuroides* and *S. bicolor* genomes.** Circos analysis of *E. ophiuroides* and *S. bicolor* genome characteristics. a: gene density; b, LTR density; c, *athila* density; d, *del* density.


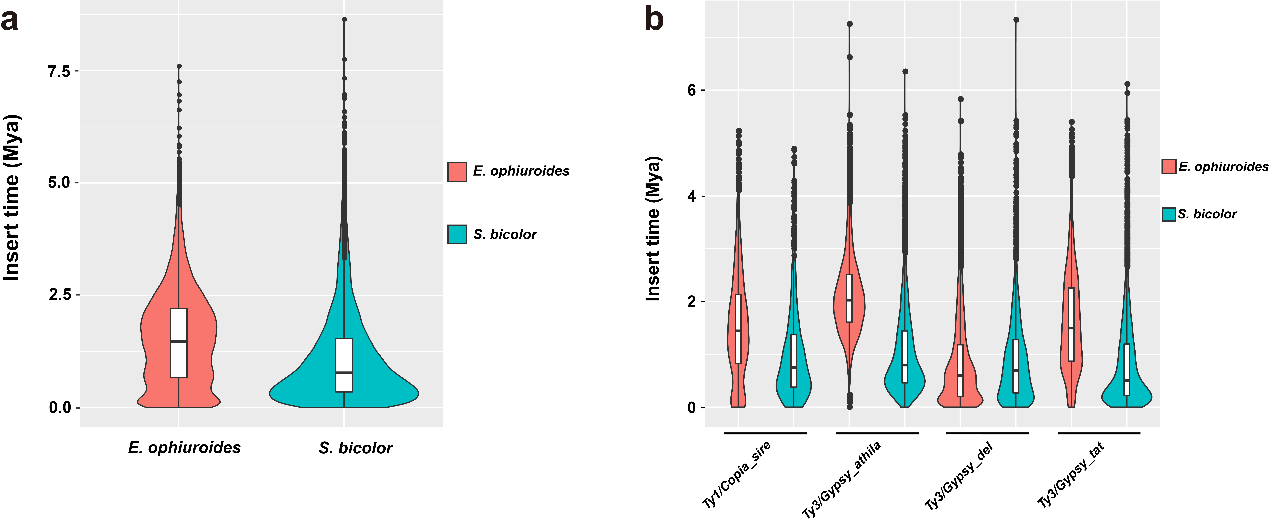


**Supplementary Fig. 6. Analyses of LTR insert times between the *E. ophiuroides* and *S. bicolor* genomes. (a)** LTR retrotransposon insert times in *E. ophiuroides* and *S. bicolor*. **(b)** The insertion times of *Ty3/Gypsy* and *Ty1/Copia* subfamilies in *E. ophiuroides* and *S. bicolor*.


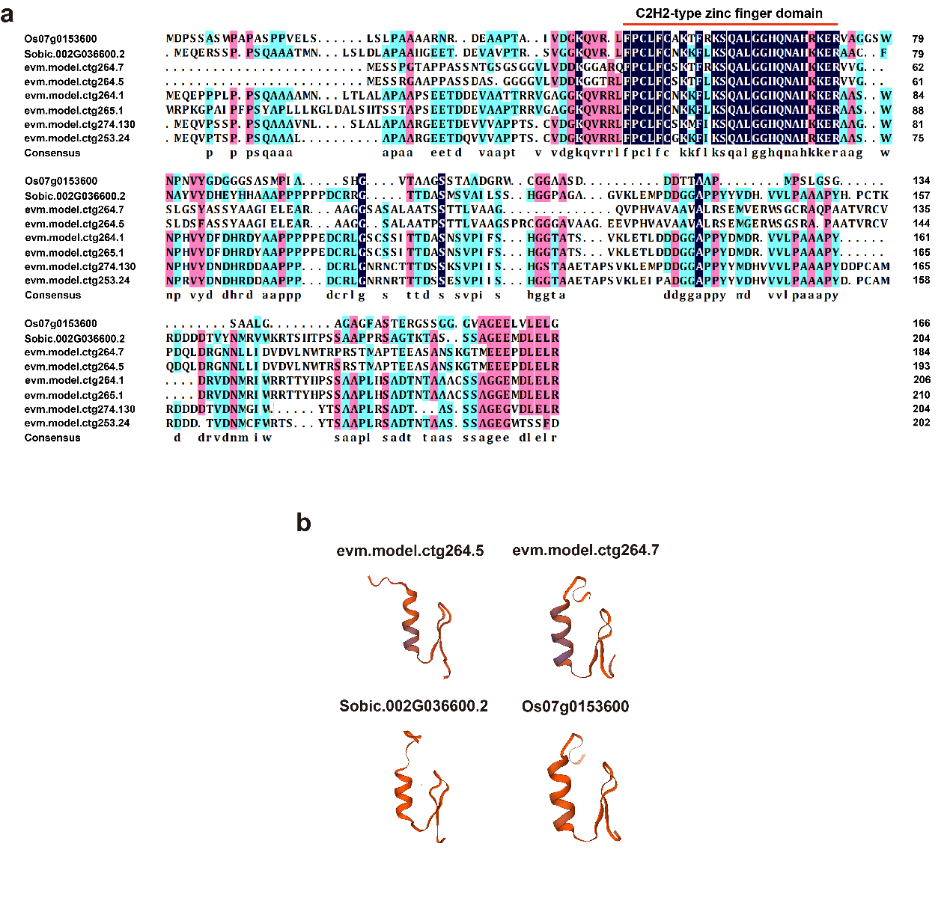


**Supplementary Fig. 7. Protein alignment and structure predictions for *PROG* genes. (a)** Protein alignment of candidate *PROG* genes in the yellow area in **Fig. 4b**. Single underlining in red indicates a conserved C2H2-type zinc-finger domain. Black represents 100% identity, red represents 75% identity, and blue represents 50% identity. **(b)** Protein structure predictions of *PROG* genes in rice, sorghum and centipedegrass.

**Supplementary Table 1 Primer sequences**

| **Gene** | **Forward primer** | **Reverse primer** |
| --- | --- | --- |
| **EoPROG1** | TCGGAGATGGTGGAGAGGT | TCGATCAGCAGGTTGTTGC |
| **EoPROG2** | GGTCGGAGATGGGGGAAA | TCGATCAGCAGGTTGTTG |
| **evm.model.ctg253.24** | CCCTGGATTCGTCCTTCACATA | CACAGGGCAAGCATATTACTGC |
| **evm.model.ctg264.1** | TTCTAACTCCGTCCCGATCTTC | GGTAGGTCGTCCTTCTCCAGAT |
| **evm.model.ctg265.1** | TTCTAACTCCGTCCCGATCTTC | GGTAGGTCGTCCTTCTCCAGAT |
| **evm.model.ctg274.130** | CTAAGTCCGTCCCGATCATCTC | AGAGGTGTACCAGATGCCCATG |
| **EoActin** | GCACGGAATCGTCAGCAA | CCCTCGTAGATGGGGACAGT |
| **Hyg** | CGACAGCGTCTCCGACCTGAT | AGATGTTGGCGACCTCGTATTG |
| **AtAct2** | TTCGTTTTGCGTTTTAGTCCC | GGGAACAAAAGGAATAAAGAGGC |

**Supplementary Table 2 Statistics of the survey of the *E. ophiuroides* genome**

| **K-mer** | **K-mer number** | **K-mer Depth** | **Genome Size (Mb)** | **Revised Genome Size (Mb)** | **Heterozygous Ratio (%)** | **Repeat (%)** |
| --- | --- | --- | --- | --- | --- | --- |
| 17 | 38,347,089,808 | 44 | 871.52 | 856.12 | 0.76 | 59.87 |

**Supplementary Table 3 Statistics of sequencing data for the *E. ophiuroides* genome**

| **Library type** | **Insert size** | **Total data (G)** | **Sequence coverage (X)** |
| --- | --- | --- | --- |
| Illumina reads | 350 bp | 111.18 | 130.47 |
| Pacbio reads | 42 Kb | 89.46 | 104.98 |
| 10X Genomics | - | 107.23 | 125.84 |
| BioNano | - | 108.37 | 124.93 |
| Hi-C | - | 106.45 | 122.72 |
| Total | - | 307.87 | 361.29 |

**Supplementary Table 4 Assessment of sequence consistency for the *E. ophiuroides* genome**

|  |  | **Percentage** |
| --- | --- | --- |
| Reads | Mapping rate (%) | 98.87 |
| Genome | Average sequencing depth | 108.91 |
|  | Coverage (%) | 99.78 |
|  | Coverage at least 4X (%) | 99.66 |
|  | Coverage at least 10X (%) | 99.49 |
|  | Coverage at least 20X (%) | 99.16 |

**Supplementary Table 5 Quality assessment of the *E. ophiuroides* genome**

| **Type** | **Number** | **Percent (%)** |
| --- | --- | --- |
| Complete BUSCOs (C) | 1374 | 95.4 |
| Complete and single-copy BUSCOs (S) | 1273 | 88.4 |
| Complete and duplicated BUSCOs (D) | 101 | 7.0 |
| Fragmented BUSCOs (F) | 19 | 1.3 |
| Missing BUSCOs (M) | 47 | 3.3 |
| Total BUSCO groups searched | 1440 | 100 |

**Supplementary Table 6 Chromosome clustering of the *E. ophiuroides* genome.**

| **Group** | **Number** | **Length (bp)** |
| --- | --- | --- |
| Chr1 | 52 | 114,806,598 |
| Chr2 | 39 | 110,364,709 |
| Chr3 | 36 | 106,363,105 |
| Chr4 | 42 | 97,333,449 |
| Chr5 | 26 | 86,051,941 |
| Chr6 | 31 | 82,208,441 |
| Chr7 | 28 | 72,060,952 |
| Chr8 | 32 | 68,685,498 |
| Chr9 | 14 | 65,489,550 |
| Total | 300 | 803,364,243 (92.61%) |

**Supplementary Table 7 Statistics of repetitive sequences of the *E. ophiuroides* genome**

| **Type** | **Repeat Size (bp)** | **% of genome** |
| --- | --- | --- |
| TRF | 28,616,952 | 3.30 |
| RepeatMasker | 500,501,482 | 57.70 |
| RepeatProteinMask | 176,088,907 | 20.30 |
| Total | 536,065,955 | 61.80 |

**Supplementary Table 8 Statistics of TE classification**

|  | **Denovo+Repbase** | | **TE Proteins** | | **Combined TEs** | |
| --- | --- | --- | --- | --- | --- | --- |
|  | **Length (bp)** | **% in Genome** | **Length (bp)** | **% in Genome** | **Length (bp)** | **% in Genome** |
| DNA | 24,080,143 | 2.7760 | 21,343,299 | 2.4605 | 36,028,098 | 4.1534 |
| LINE | 4,650,951 | 0.5362 | 15,159,446 | 1.7476 | 17,136,270 | 1.9755 |
| SINE | 27,871 | 0.0032 | 0 | 0.0000 | 27,871 | 0.0032 |
| LTR | 462,954,018 | 53.3708 | 140,053,645 | 16.1458 | 470,691,745 | 54.2628 |
| *Ty1/copia* | 84,006,991 | 9.6846 | 36,829,806 | 4.2459 | 91,065,365 | 10.4983 |
| *Ty3/gypsy* | 349,319,756 | 40.2707 | 102,405,631 | 11.8056 | 361,436,178 | 41.6675 |
| Other | 29,627,271 | 3.4155 | 818,208 | 0.0943 | 18,190,202 | 2.0970 |
| Unknow | 6,040,989 | 0.6964 | 14,975 | 0.0017 | 6,055,964 | 0.6982 |
| Total | 500,501,482 | 57.6994 | 176,088,907 | 20.3001 | 528,486,578 | 60.9256 |

**Supplementary Table 9 Statistics of genetic structures**

| **Species** | **Number** | **Average gene length (bp)** | **Average CDS length (bp)** | **Average exons per gene** | **Average exon length (bp)** | **Average intron length (bp)** |
| --- | --- | --- | --- | --- | --- | --- |
| *Eremochloa ophiuroides* | 36,572 | 3278.42 | 1144.64 | 4.48 | 255.43 | 612.94 |
| *Sorghum bicolor* | 34,129 | 3146.86 | 1227.98 | 5.09 | 241.13 | 468.86 |
| *Setaria viridis* | 35,214 | 2454.35 | 1166.55 | 4.55 | 256.34 | 362.67 |
| *Hordeum vulgare* | 23,853 | 2021.46 | 1051 | 3.87 | 271.77 | 338.46 |
| *Brachypodium distachyon* | 26,480 | 2853.91 | 1282.07 | 5.01 | 255.86 | 391.9 |
| *Zea mays* | 38,985 | 3488.05 | 1185.11 | 4.95 | 239.66 | 583.76 |
| *Oryza sativa* | 34,227 | 2205.44 | 1003.17 | 3.83 | 262.06 | 425.13 |
| *Zoysia japonica* | 65,252 | 2574.02 | 902 | 3.93 | 229.72 | 571.33 |
| *Triticum_aestivum* | 102,941 | 3068.81 | 1261.54 | 4.49 | 281.1 | 518.17 |

**Supplementary Table 10 Statistics of noncoding RNA**

| Type | Number | Average length (bp) | Total length (bp) | % of genome |
| --- | --- | --- | --- | --- |
| miRNA | 2856 | 176.2766106 | 503,446 | 0.0580 |
| tRNA | 616 | 74.90746753 | 46,143 | 0.0053 |
| rRNA | 231 | 185.2727273 | 42,798 | 0.0049 |
| 18S | 27 | 742.037037 | 20,035 | 0.0023 |
| 28S | 31 | 141.8387097 | 4,397 | 0.0005 |
| 5.8S | 10 | 150.1 | 1,501 | 0.0002 |
| 5S | 163 | 103.4662577 | 16,865 | 0.0019 |
| snRNA | 5174 | 108.8735988 | 563,312 | 0.0649 |
| CD-boc | 4968 | 107.4722222 | 533,922 | 0.0616 |
| HACA-box | 56 | 129.4642857 | 7,250 | 0.0008 |
| splicing | 150 | 147.6 | 22,140 | 0.0026 |

**Supplementary Table 11 Statistics of gene annotation**

|  | Database | Annotated Number | Annotated Percent (%) |
| --- | --- | --- | --- |
| Annotation | NR | 34,089 | 93.2 |
|  | Swiss-Prot | 24,994 | 68.3 |
|  | KEGG | 23,850 | 65.2 |
|  | InterPro-All | 27,784 | 76 |
|  | InterPro-Pfam | 25,726 | 70.3 |
|  | InterPro-GO | 18,935 | 51.8 |
| Total | Annotated | 34,161 | 93.4 |
|  | Gene | 36,572 | - |

**Supplementary Table 12 Selection of species**

| Species | Name | Gene number |
| --- | --- | --- |
| *E. ophiuroides* | *Eremochloa_ophiuroides* | 36,572 |
| *A. tauschii* | *Aegilops_tauschii* | 42,826 |
| *A. thaliana* | *Arabidopsis_thaliana* | 26,869 |
| *B. distachyon* | *Brachypodium_distachyon* | 26,480 |
| *H. vulgare* | *Hordeum_vulgare* | 39,734 |
| *O. sativa* | *Oryza_sativa* | 34,227 |
| *P. hallii* | *Panicum_hallii* | 36,958 |
| *S. italica* | *Setaria_italica* | 33,439 |
| *S. viridis* | *Setaria_viridis* | 34,936 |
| *Z. mays* | *Zea_mays* | 38,985 |
| *S. bicolor* | *Sorghum_bicolor* | 34,129 |
| *Z. japonica* | *Zoysia_japonica* | 59,271 |
| *T. aestivum* | *Triticum_aestivum* AA | 32,147 |
| *T. aestivum* | *Triticum_aestivum* BB | 28,640 |
| *T. aestivum* | *Triticum_aestivum* DD | 32,389 |

**Supplementary Table 13 Gene numbers of ROS, flavonoid-related and R gene families in *E. ophiuroides* and *S. bicolor***

| **Family** | **Gene ID** | **Gene number** | |
| --- | --- | --- | --- |
|  |  | ***E. ophiuroides*** | ***S. bicolor*** |
| ROS-related gene | *FLS2* | 3 | 3 |
|  | *RBOH* | 12 | 10 |
| Flavonoid-related gene | *4CL* | 24 | 21 |
|  | *ACC1* | 2 | 2 |
|  | *ANR* | 36 | 45 |
|  | *C4H* | 94 | 75 |
|  | *CHI* | 3 | 2 |
|  | *CHS* | 16 | 36 |
|  | *DFR* | 38 | 47 |
|  | *F3H* | 75 | 78 |
|  | *F3’H* | 204 | 186 |
|  | *FLS* | 73 | 84 |
|  | *LAC15* | 23 | 31 |
|  | *LDOX* | 41 | 51 |
|  | *PAL* | 6 | 8 |
|  | *UFGT* | 70 | 51 |
| R gene | *NBS* | 597 | 337 |

**Supplementary Table 14 Numbers of different full-length LTR families in *E. ophiuroides* and *S. bicolor***

| **Family** | **Subfamily** | ***E. ophiuroides*** | ***S. bicolor*** |
| --- | --- | --- | --- |
| *Gypsy* | *athila* | 5,218 | 6,681 |
|  | *del* | 5,436 | 2,775 |
|  | *tat* | 2,761 | 4,422 |
|  | other | 843 | 948 |
| *Copia* | *sire* | 2,180 | 1,080 |
|  | other | 1,327 | 1,118 |
| other | | 67 | 12 |
| unknown | | 3,494 | 2,932 |
| Total | | 21,326 | 19,968 |

**Supplementary Table 16 *PROG1* and its homologous genes in *O. sativa*, *S. bicolor* and *E. ophiuroides***

| **Species** | **Gene ID** | **Chromosome** |
| --- | --- | --- |
| *O. sativa* | Os07g0153600 | chromosome 7 |
|  | Os03g0786400 | chromosome 3 |
|  | Os01g0132766 | chromosome 1 |
|  | Os01g0512700 | chromosome 1 |
|  | Os09g0430600 | chromosome 9 |
| *S. bicolor* | Sobic.002G036600.2 | chromosome 2 |
|  | Sobic.002G036500.1 | chromosome 2 |
|  | Sobic.002G036800.1 | chromosome 2 |
|  | Sobic.008G173200.1 | chromosome 8 |
|  | Sobic.008G173300.1 | chromosome 8 |
|  | Sobic.003G084400.2 | chromosome 3 |
| *E. ophiuroides* | evm.model.ctg301.23 | chromosome 1 |
|  | evm.model.ctg290.63 | chromosome 1 |
|  | evm.model.ctg187.144 | chromosome 2 |
|  | evm.model.ctg264.7 | chromosome 2 |
|  | evm.model.ctg264.1 | chromosome 2 |
|  | evm.model.ctg264.5 | chromosome 2 |
|  | evm.model.ctg264.3 | chromosome 2 |
|  | evm.model.ctg265.1 | chromosome 2 |
|  | evm.model.ctg274.130 | chromosome 2 |
|  | evm.model.ctg253.24 | chromosome 2 |
|  | evm.model.ctg434.64 | chromosome 3 |
|  | evm.model.ctg464.152 | chromosome 3 |
|  | evm.model.ctg421.94 | chromosome 3 |
|  | evm.model.ctg699.73 | chromosome 6 |
