# Supplementary material for: A high-quality chromosome-scale assembly of the centipedegrass [Eremochloa ophiuroides (Munro) Hack.] genome provides insights into chromosomal structural evolution and prostrate growth habit
Source: Hortic Res. 2021 Sep 1;8:201. doi: 10.1038/s41438-021-00636-6 (PMC8408263; doi:10.1038/s41438-021-00636-6)
Supplement: Supplementary file 3 — Supplementary method [file 41438_2021_636_MOESM3_ESM.docx]

**Supplementary method**

**Genome sequencing and assembly**

Leaf tissue of *E. ophiuroides* ‘Ganbei’ was collected for DNA extraction with the DNeasy Plant Mini Kit (QIAGEN) and used for Illumina, PacBio, BioNano and Hi-C library construction. An Illumina library with an insert size of ~350 bp was prepared using Illumina’s Genomic DNA Sample Preparation kit and sequenced on the Illumina HiSeq 2500 platform. For the PacBio Sequel analysis, SMRTbell TM libraries were prepared according to the manufacturer’s protocol of the sequencing platform. A 15 kb DNA SMRTbell library was constructed and sequenced on a Pacbio Sequel II platform. We generated 89.46 Gb (approximately 105-fold depth) of raw data. The DNA samples employed for BioNano library construction were extracted with a BioNano Sample Prep kit (RE-014-05) and digested with a Bionano NLRS DNA Labeling Kit (BioNano, [https://bionanogenomics.com](https://bionanogenomics.com/zh/bionano-genomics/)). The BioNano library was sequenced on the Saphyr platform. High molecular weight genomic DNA extraction, sample indexing, and generation of partition barcoded libraries were performed according to the 10× Genomics (Pleasanton, CA, USA) Chromium Genome User Guide and sequenced on the Illumina HiSeq X Ten platform. The Hi-C library was prepared using the NEBNext Ultra II DNA Library Prep Kit for Illumina (NEB, England) and sequenced on the Illumina NovaSeq PE150 platform. All DNA extraction and sequencing procedures were performed by the Novogene Company (Tianjin, China) (<http://www.novogene.com/>).

The PacBio reads were used for *de novo* assembly, and the FALCON-Unzip assembler (<https://github.com/PacificBiosciences/FALCON>)^1^ was used to develop an initial set of scaffolds and subsequently polished using Quiver consensus caller^2^. The assemblies were further polished with Illumina data using Pilon v1.22^3^ to correct indel errors associated with homopolymer repeats in the PacBio data. We further removed the redundant contigs by Purge Haplotigs (v1.10)^4^. This produced 1,047 scaffolds, with a scaffold N50 of 1.86 Mb, and a total genome size of 849.64 Mb. To order and orient these scaffolds into longer blocks, we obtained sequence data from libraries generated using the 10× Genomics platform. In total, 125.84× barcoded sequence coverage was obtained. We used the program fragScaff^5^ to achieve this scaffolding, which increased the scaffold N50 of our assembly to 3.66 Mb.

These scaffolds were processed for hybrid assembly by using high-resolution optical mapping (BioNano Genomics Irys) data from the same accessions. BioNano data were first filtered based on molecule length, mapping rate and label density using BioNano Solve (https://bionanogenomics.com/wp-content/uploads/2017/10/30182-Bionano-Tools-Installation-Guide.pdf). Non-haplotype *de novo* assembly was performed in BioNano Solve using filtered high-quality BNX files based on the Overlap-Layout-Consensus paradigm. The 234,215 filtered DLE-1 molecules (N50 length 0.236 Mb) produced 2,356 genome maps with an N50 of 1.05 Mb for a total map length of 1,429 Mb. The super scaffolding was performed using parameter ‘-T 1e-8’ after manual inspection of the quality of the scaffolds. The super scaffolding using the optical map generated a genome assembly consisting of 363 scaffolds with N50 to 6.1M.

To anchor scaffolds onto pseudochromosomes, HiCUP v0.6.1 was used to map and process the reads from the Hi-C library^6^. Both reads from a single pair that uniquely mapped to the assembly were retained for downstream filtration. Invalid pairs generated from contiguous sequences, circularization, dangling ends, internal fragments, religation, PCR duplication, and fragments of the wrong size were removed. To validate the correction of the Hi-C scaffolding results at the pseudochromosome level, we constructed an interaction matrix with clean reads from the Hi-C library using HiC-Pro^7^. The genome was divided into bins of equal sizes (500 k), and the number of contacts between each pair of reported bins was determined. A contact map plotted with HiCPlotter confirmed the genome structure and quality^8^. To assess genome assembly quality, the Benchmarking Universal Single-Copy Orthologs (BUSCO) v3 tool (http://busco.ezlab.org/)^9^ was used with single-copy orthologous genes. The sequence consistency and quality of the assembled genome were evaluated using the Burrows-Wheeler Aligner (BWA)^10^ and BUSCO^11^.

**Genome prediction and annotation**

Genome prediction and annotation mainly included repeat sequence prediction, gene annotation and noncoding RNA (ncRNA) prediction. Repeat sequences were predicted through homology searches in the Repbase database (http://www.girinst.org/repbase) by using RepeatMaster and RepeatProteinMask software (<http://www.repeatmasker.org/>)^12^. Additionally, *de novo* library construction was performed using three software programs: LTR_FINDER (http://tlife.fudan.edu.cn/ltr_finder/), RepeatScout (http://www.repeatmasker.org/), and RepeatModeler (<http://www.repeatmasker.org/RepeatModeler/html/>). Then, the *de novo* annotation of repeat elements was performed with Repeatmasker^13^. The DNA substitution rates (K) between the 5’ and 3’ LTRs were calculated using baseml in the PAML package^14^. The insertion times of the LTRs were estimated using the formula K/2r (r = 1.3 × 10^-8^), considering a higher substitution rate in intergenic regions than in coding regions.

Gene structure annotation was carried out with a combined prediction method including homology, *de novo*, and RNA transcript data. For homology prediction, a set of protein-coding sequences from *B. distachyon*, *H. vulgare*, *O. sativa*, *S. viridis*, *Z. mays*, *S. bicolor*, *Z. japonica* and *T. aestivum* was mapped to the *E. ophiuroides* genome assembly with BLAST (<http://blast.ncbi.nlm.nih.gov/Blast.cgi>) (E value ≤ 1e^-5^). GeneWise (http://www.ebi.ac.uk/~birney/wise2/) was previously used to predict the gene structure of each hit^15^. Augustus (<http://bioinf.uni-greifswald.de/augustus/>), GlimmerHMM (<http://ccb.jhu.edu/software/glimmerhmm/>) and SNAP (<http://homepage.mac.com/iankorf/>) were used for *de novo* gene structure prediction. RNA-seq data from roots, stems, leaves, nodes and spikes were also used to identify gene structures with BLAT (http://genome.ucsc.edu/cgi-bin/hgBlat). In total, 45,472 primitive gene models predicted from the above three prediction methods were corrected with RNA-seq data and integrated into a nonredundant and more complete gene set by using EVidenceModeler (EVM, <http://evidencemodeler.sourceforge.net/>)^16^. Furthermore, the transcriptome assembly results were combined, and the Program to Assemble Spliced Alignment (PASA, <http://pasa.sourceforge.net/>) was used to correct the EVM annotation results by adding information on untranslated regions and alternative splicing^16^. Finally, a gene set containing 36,572 gene models was obtained.

Gene function annotation was performed via BLASTP (E value ≤ 1e^-5^) searches against the NR (https://www.ncbi.nlm.nih.gov/), SwissProt (http://www.UniProt.org/), KEGG (http://www.genome.jp/kegg/) and InterPro (<https://www.ebi.ac.uk/interpro/>) protein databases. Functional domains were identified in the InterPro (https://www.ebi.ac.uk/interpro/) and Pfam (http://pfam.xfam.org/) databases using InterProScan^17^. The Gene Ontology (GO) terms for each gene were identified from the GO database^18^.

The noncoding RNA annotations included tRNAs, rRNAs, miRNAs and snRNAs. On the basis of structural characteristics, tRNAscan-SE (<http://lowelab.ucsc.edu/tRNAscan-SE/>) was used to search the tRNA sequences in the genome assembly of *E. ophiuroides*. Because of the high conservation of rRNA sequences among plants, the rRNAs of *E. ophiuroides* were identified by BLAST searches in related species. The miRNA and snRNA sequences were predicted by applying INFERNAL (<http://infernal.janelia.org/>) against the Rfam database (v12.0).

**References**

1 Chin CS, Peluso P, Sedlazeck FJ *et al*. Phased diploid genome assembly with single-molecule real-time sequencing. *Nat Methods* 2016; 13: 1050-1054.

2 Chin CS, Alexander DH, Marks P *et al*. Nonhybrid, finished microbial genome assemblies from long-read SMRT sequencing data. *Nat Methods* 2013; 10, 563-569.

3 Walker BJ, Abeel T, Shea T *et al*. Pilon: an integrated tool for comprehensive microbial variant detection and genome assembly improvement. *Plos One* 2014; 9: e112963.

4 Roach MJ, Schmidt SA, Borneman AR. Purge Haplotigs: allelic contig reassignment for third-gen diploid genome assemblies. *BMC Bioinformatics* 2018; 19, 460.

5 Adey A, Kitzman JO, Burton JN *et al.* In vitro, long-range sequence information for de novo genome assembly via transposase contiguity. *Genome Res* 2014; 24, 2041-2049.

6 Wingett S, Ewels P, Furlan-Magaril M *et al*. HiCUP: pipeline for mapping and processing Hi-C data. *F1000Res* 2015; 4.

7 Servant N, Varoquaux N, Lajoie BR *et al*. HiC-Pro: an optimized and flexible pipeline for Hi-C data processing. *Genome Biol* 2015; 16: 259.

8 Akdemir KC, Chin L. HiCPlotter integrates genomic data with interaction matrices. *Genome Biol* 2015; 16: 198.

9 Simao FA, Waterhouse RM, Ioannidis P, Kriventseva EV, Zdobnov EM. BUSCO: assessing genome assembly and annotation completeness with single-copy orthologs. *Bioinformatics* 2015; 31: 3210-3212.

10 Li H, Durbin R. Fast and accurate short read alignment with Burrows-Wheeler Transform. *Bioinformatics* 2009; 25: 1754-60.

11 Simão FA, Waterhouse RM, Ioannidis P, Kriventseva EV, Zdobnov EM. BUSCO: assessing genome assembly and annotation completeness with single-copy orthologs. *Bioinformatics* 2015; 31: 3210-3212.

12 Bao W, Kojima KK, Kohany O. Repbase Update, a database of repetitive elements in eukaryotic genomes. *Mobile DNA-UK* 2015; 6: 11.

13 Price AL, Jones NC, Pevzner PA. *De novo* identification of repeat families in large genomes. *Bioinformatics* 2005; 21: 351-358.

14 Zhao K, Henderson E, Bullard K, Oberste MS, Burns CC, Jorba J. PoSE: visualization of patterns of sequence evolution using PAML and MATLAB. *BMC Bioinformatics* 2018; 19(11).

15 Birney E, Clamp M, Durbin R. GeneWise and genomewise. *Genome Res* 2004; 14: 988-995.

16 Haas BJ, Salzberg SL, Zhu W *et al*. Automated eukaryotic gene structure annotation using EVidenceModeler and the program to assemble spliced alignments. *Genome Biol* 2008; 9: R7.

17 Jones P, Binns D, Chang HY *et al*. InterProScan 5: genome-scale protein function classification. *Bioinformatics* 2014; 30: 1236-1240.

18 Dimmer EC, Huntley RP, Alam-Faruque Y *et al*. The UniProt-GO annotation database in 2011. *Nucleic Acids Res* 2011; 40: 565-570.
